# *De novo* DNA methylation controls neuronal maturation during adult hippocampal neurogenesis

**DOI:** 10.15252/embj.2020107100

**Published:** 2021-08-02

**Authors:** Sara Zocher, Rupert W Overall, Gabriel Berdugo‐Vega, Nicole Rund, Anne Karasinsky, Vijay S Adusumilli, Christina Steinhauer, Sina Scheibenstock, Kristian Händler, Joachim L Schultze, Federico Calegari, Gerd Kempermann

**Affiliations:** ^1^ German Center for Neurodegenerative Diseases (DZNE) Dresden Germany; ^2^ Center for Regenerative Therapies Dresden (CRTD) Technische Universität Dresden Dresden Germany; ^3^ PRECISE Platform for Single Cell Genomics and Epigenomics German Center for Neurodegenerative Diseases University of Bonn Bonn Germany; ^4^ Present address: Max Planck Institute of Neurobiology Martinsried Germany

**Keywords:** adult neurogenesis, DNA methylation, Dnmt3a, hippocampus, neuron maturation, Chromatin, Epigenetics, Genomics & Functional Genomics, Neuroscience

## Abstract

Adult neurogenesis enables the life‐long addition of functional neurons to the hippocampus and is regulated by both cell‐intrinsic molecular programs and behavioral activity. *De novo* DNA methylation is crucial for embryonic brain development, but its role during adult hippocampal neurogenesis has remained unknown. Here, we show that *de novo* DNA methylation is critical for maturation and functional integration of adult‐born neurons in the mouse hippocampus. Bisulfite sequencing revealed that *de novo* DNA methyltransferases target neuronal enhancers and gene bodies during adult hippocampal neural stem cell differentiation, to establish neuronal methylomes and facilitate transcriptional up‐regulation of neuronal genes. Inducible deletion of both *de novo* DNA methyltransferases Dnmt3a and Dnmt3b in adult neural stem cells did not affect proliferation or fate specification, but specifically impaired dendritic outgrowth and synaptogenesis of newborn neurons, thereby hampering their functional maturation. Consequently, abolishing *de novo* DNA methylation modulated activation patterns in the hippocampal circuitry and caused specific deficits in hippocampus‐dependent learning and memory. Our results demonstrate that proper establishment of neuronal methylomes during adult neurogenesis is fundamental for hippocampal function.

## Graphical abstract

Ablation of DNA methyltransferases Dnmt3a/b shows that *de novo* DNA methylation is not needed for adult neural stem cell proliferation and fate determination, but for morphological and functional maturation of adult‐born neurons in the hippocampus.

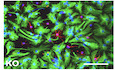

## Introduction

The dentate gyrus of the hippocampus harbors a stem cell population that generates new neurons throughout life (Bond *et al*, 2015; Kempermann *et al*, 2015). Adult‐born neurons are critical for hippocampus‐dependent cognitive flexibility and impairments in their production have been implicated in age‐related cognitive decline and neuropsychiatric disorders such as depression (Anacker *et al*, 2018; Toda *et al*, 2019; Berdugo‐Vega *et al*, 2020). Adult hippocampal neural stem and progenitor cells (NSPCs) originate from a specific population of embryonic NSPCs, which is highly proliferative during development but transitions to a quiescent state postnatally once the hippocampus is formed (Berg *et al*, 2019). In stark contrast to embryonic NSPCs, the activity of adult hippocampal NSPCs is environmentally regulated, as both their recruitment from quiescence and the survival of their neuronal progeny are dependent on behavioral activity (Kempermann *et al*, 1997; Adusumilli *et al*, 2021). During adult hippocampal neurogenesis, cell‐extrinsic signals are integrated with cell‐intrinsic molecular programs to orchestrate NSPC proliferation, differentiation, and neuronal maturation (Aimone *et al*, 2014; Vicidomini *et al*, 2020). Although a number of transcription factors controlling this process have been identified (Beckervordersandforth *et al*, 2015; Mukherjee *et al*, 2016; Schäffner *et al*, 2018), the epigenetic machinery which enables the molecular changes required for the generation of functional neurons from NSPCs in the adult hippocampus has remained elusive.

DNA methylation is crucial for mammalian development, where it controls lineage commitment and stem cell differentiation (Mohn & Schübeler, 2009; Smith & Meissner, 2013). Studying the DNA methylation dynamics during embryonic neurogenesis or *in vitro* neural precursor cell (NPC) differentiation has previously provided insight into the gene‐regulatory mechanisms that drive NSPC proliferation and neuronal fate specification (Mohn *et al*, 2008; Xie *et al*, 2013; Ziller *et al*, 2015; Luo *et al*, 2016; Ziller *et al*, 2018; Noack *et al*, 2019). However, the DNA methylation patterns of adult hippocampal NSPCs and their changes during adult neurogenesis had remained unknown. Since adult hippocampal NSPCs differ from embryonic NSPCs in terms of transcriptomic signatures, regulation, and extracellular environment (Urbán & Guillemot, 2014; Berg *et al*, 2019), the function of DNA methylation changes during hippocampal neurogenesis cannot be inferred from studies of embryonic neurogenesis. Previous reports demonstrated that perturbing the demethylating enzymes Tet1 and Tet2 or the methylation readers Mbd1 and Mecp2 impaired adult hippocampal neurogenesis at the level of NSPC proliferation, differentiation, or neuronal maturation (Smrt *et al*, 2007; Zhang *et al*, 2013; Jobe *et al*, 2017; Li *et al*, 2017; Gontier *et al*, 2018). These studies already suggested that DNA methylation controls aspects of adult hippocampal neurogenesis; however, the specific role of *de novo* DNA methylation during adult neurogenesis had not been resolved.

DNA methylation is catalyzed by the activity of DNA methyltransferases. Whereas Dnmt1 maintains global DNA methylation patterns during replication, *de novo* DNA methyltransferases Dnmt3a and Dnmt3b catalyze the addition of new methyl groups to previously non‐methylated genomic regions (Ma *et al*, 2010). *De novo* DNA methyltransferases are crucial for embryonic and postnatal brain development and also for memory formation in adulthood (Nguyen *et al*, 2007; Wu *et al*, 2010; Gulmez Karaca *et al*, 2020; Odell *et al*, 2020). However, their specific role during adult hippocampal neurogenesis has been as yet unknown. Previous studies suggested that, in NSPCs derived from *Dnmt3a*‐deficient embryonic stem cells (ESCs), astrogliogenesis was increased at the expense of neurogenesis (Wu *et al*, 2010, 2012; Ziller *et al*, 2018). However, because inducible *Dnmt3a* knock‐out models had not been used, it remained unknown whether a neurogenic fate of NSPCs is pre‐defined by DNA methylation patterns established during early development or whether it is specified by further *de novo* DNA methylation in the course of terminal neuronal differentiation.

Here, we provide a comprehensive characterization of the function of *de novo* DNA methylation during *in vitro* and *in vivo* adult hippocampal neurogenesis, including its relevance for activity‐dependent activation in the hippocampal circuitry and consequently for animal behavior and cognition. Using an inducible, NSPC‐specific knock‐out model of *de novo* DNA methyltransferases, we demonstrate the specific requirement of neurogenesis‐associated *de novo* DNA methylation for the maturation and functional integration of adult‐born neurons into the hippocampus.

## Results

### Neuronal differentiation of adult hippocampal NPCs is associated with focal DNA methylation changes

**Figure 1:**
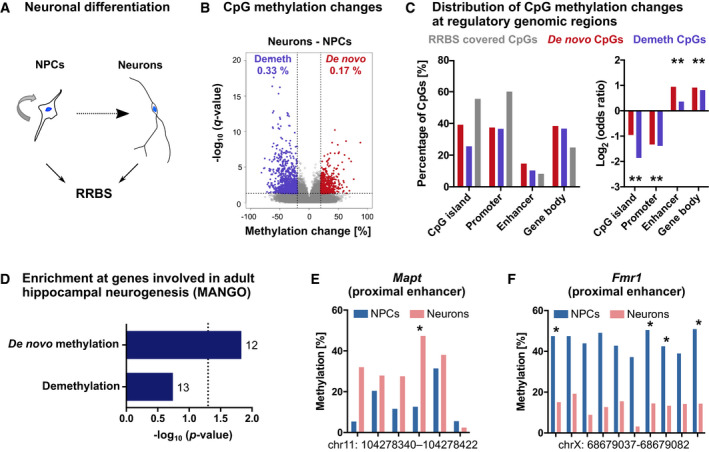
DNA methylation changes during neuronal differentiation of adult mouse hippocampal neural precursor cells AAdult mouse hippocampal neural precursor cells (NPCs) were differentiated into neurons. DNA methylation patterns of NPCs and purified neurons were analyzed by reduced representation bisulfite sequencing (RRBS; *n* = 3 cultures).BVolcano plots depicting CpG methylation changes between cell populations. Significantly differentially methylated CpGs with *q* < 0.05 and absolute methylation differences > 20% are highlighted in violet (demethylated CpGs—Demeth) and red (*de novo* methylated CpGs—*De novo*). Percentages of differentially methylated CpGs among all CpGs covered by RRBS are indicated with the respective color.CDifferentially methylated CpGs were depleted at promoters and CpG islands but enriched at gene bodies and enhancers compared to all CpGs covered by RRBS. Asterisks (*) indicate genomic regions with FDR adjusted *P* < 0.05 (linear regression).DGenes with known role in adult hippocampal neurogenesis as annotated in the Mammalian Adult Neurogenesis Gene Ontology (MANGO; 359 annotated genes) were significantly enriched among *de novo* methylated genes (494 genes; *P*‐values are from hypergeometric test). Numbers of overlapping genes are indicated at the respective bars.E, FCandidate gene regions with neurogenesis‐associated *de novo* methylation (E) and demethylation (F). Depicted are mean methylation levels per group at individual CpGs within the specified genomic regions. Asterisks (*) indicate significantly differentially methylated CpGs with *q* < 0.05 (logistic regression with multiple testing correction using SLIM method) and methylation difference > 20%.

To first investigate whether DNA methylation patterns are dynamic during adult hippocampal neurogenesis in mice, we isolated NPCs from the adult mouse hippocampus, differentiated them *in vitro* and isolated differentiated neurons by fluorescence‐activated cell sorting (FACS; Fig 1A and Appendix Fig S1A and B). We performed reduced representation bisulfite sequencing (RRBS; Boyle *et al*, 2012) on NPCs and neurons and obtained DNA methylation levels of 310,453 CpGs which were sufficiently covered by RRBS in all samples. We identified 1,551 CpGs (0.50% of CpGs covered by RRBS) that significantly changed methylation during differentiation from NPCs into neurons (Fig 1B; Dataset EV1). Among these CpGs, 33.72% gained methylation (*de novo* methylated CpGs) during neuronal differentiation, while 66.28% were demethylated. Both *de novo* methylated and demethylated CpGs were depleted at promoters and CpG islands, but significantly enriched at putative enhancers and gene bodies relative to all CpGs covered by RRBS (Fig 1C). To gain first insight into a potential role of the identified DNA methylation changes in controlling adult hippocampal neurogenesis, we annotated differentially methylated CpGs to the nearest located gene and performed gene set enrichment analysis with genes listed in the Mammalian Adult Neurogenesis Gene Ontology (MANGO; Overall *et al*, 2012). We found that genes with known function in adult hippocampal neurogenesis were significantly enriched among the 494 *de novo* methylated genes (Fig 1D). Specifically, 12 *de novo* methylated genes had functional annotations in MANGO (Dataset EV2). An example of such a *de novo* methylated region is an intragenic enhancer of the gene *Mapt* (Fig 1E), which encodes the microtubule‐associated neuronal protein Tau that is required for the maturation of adult‐born neurons in the hippocampus (Pallas‐Bazarra *et al*, 2016). Loci that were demethylated during neuronal differentiation (830 genes) also comprised genes with functional annotation in MANGO, such as *Fmr1* (Fig 1F), but showed overall no significant enrichment with adult hippocampal neurogenesis‐related genes (Fig 1D). Together, these results demonstrated that there are focal DNA methylation alterations during the *in vitro* differentiation of adult hippocampal NPCs into neurons. This included the *de novo* DNA methylation of genes with known relevance in adult hippocampal neurogenesis.

### *De novo* DNA methyltransferases control neuronal differentiation of adult hippocampal NPCs *in vitro*

**Figure EV1:**
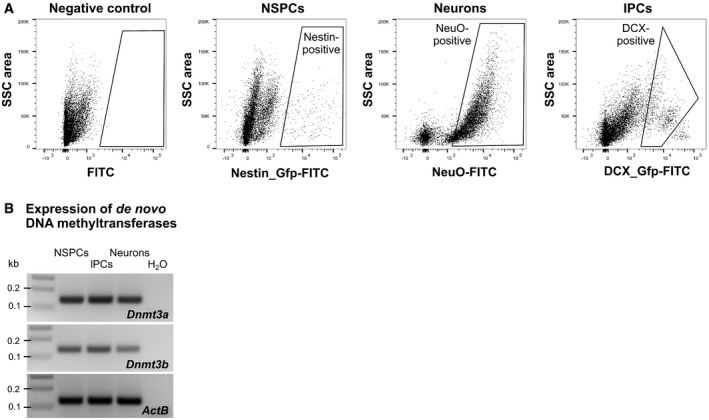
*De novo* methyltransferases are expressed in different cell stages of adult hippocampal neurogenesis FACS strategy for isolation of neural stem and early progenitor cells (NSPCs), late progenitor cells (lPCs), and neurons from the adult mouse hippocampus. After separating single cells using their forward scatter (FSC) and side scatter (SSC) properties, dead cells were excluded based on incorporation of propidium iodide. NSPCs were isolated from *Nestin*::EGFP mice and lPCs from *Dcx*::GFP mice based on Gfp intensity. Neurons were identified based on incorporation of the neuron‐specific dye NeuroFluor (NeuO).Transcript expression of *de novo* DNA methyltransferases *Dnmt3a* and *Dnmt3b* in NSPCs, lPCs, and neurons as determined by RT–PCR.

**Figure 2:**
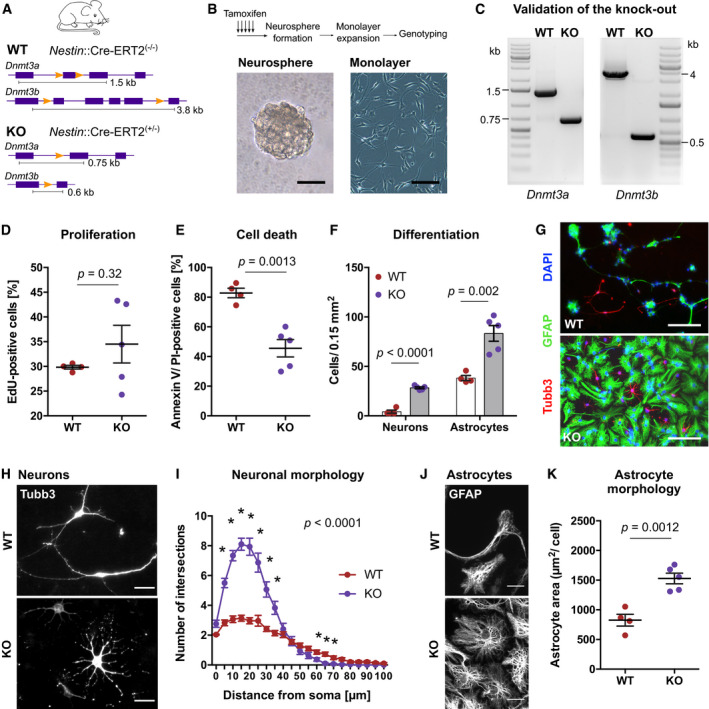
*De novo* DNA methyltransferases control neuronal differentiation from adult hippocampal NPCs *in vitro* ASchematic representation of the genomic area of *Dnmt3a* and *Dnmt3b* in wildtype (WT) mice and conditional knock‐out (KO) mice. WT mice were *Nestin*::Cre‐ERT2^(−/−)^/*Dnmt3a* ^fl/fl^/*Dnmt3b* ^fl/fl^, while KO mice were *Nestin::*Cre‐ERT2^(−/+)^/*Dnmt3a* ^fl/fl^/*Dnmt3b* ^fl/fl^.BDentate gyri from WT and KO mice were dissociated 10 days after tamoxifen administration and plated for neurosphere formation. NPC monolayer cultures were generated from single neurospheres. Representative pictures of neurosphere (left) and neurosphere‐derived adherent monolayer culture (right). Scale bars: 50 µm.CRepresentative images of polymerase chain reaction used for genotyping of NPC lines.D*Dnmt3a*/*b*‐KO did not affect NPC proliferation. Depicted are data points for every cell line with genotype means ± standard errors of the mean (SEM).EReduced percentage of dead cells (Annexin V/propidium iodide [PI] double‐positive) in KO cultures at 46 h after start of differentiation. Depicted are data points for every cell line with genotype means ± SEM.F, GNPCs from WT and KO mice differentiated into astrocytes (GFAP‐positive) and neurons (Tubb3‐positive). Increased numbers of both neurons and astrocytes were generated from KO NPCs. Depicted are data points for every cell line with genotype means ± SEM. Scale bar: 100 µm.HHigh‐magnification fluorescent image of differentiated neurons. Scale bar: 20 µm.ISholl analysis of Tubb3‐labeled WT and KO neurons (*n* = 29 neurons, WT; *n* = 30 neurons, KO). Reported *P*–value corresponds to genotype effect from two‐way ANOVA. Asterisks highlight data points with *P* < 0.05 after multiple testing adjustment (Holm method) of repeated *t*‐tests. Depicted are means ± SEM.JHigh‐magnification fluorescent image of differentiated astrocytes. Scale bar: 20 µm.KAstrocytes from KO cultures covered a larger area than WT astrocytes. Data points represent means of individual cell lines (*n* > 30 astrocytes per cell line). Depicted are data points per culture with genotype means ± SEM. Data information: Depicted *P*‐values in panels (D), (E), (F), (K) are from unpaired *t*‐test. Sample sizes: *n* = 4 cultures, WT; *n* = 5 cultures, KO. Further statistical details are reported in Appendix Table S5.

We next sought to dissect the functional role of neurogenesis‐associated *de novo* DNA methylation through the deletion of *de novo* DNA methyltransferases in NSPCs. First, we confirmed that *Dnmt3a* and *Dnmt3b* are expressed in Nestin‐positive NSPCs, Doublecortin‐positive late progenitor cells, and neurons of the adult mouse hippocampus (Fig EV1). We then generated a mouse model that enables the conditional and inducible deletion of both *de novo* DNA methyltransferases during adult hippocampal neurogenesis by crossing *Nestin*::Cre‐ERT2 mice (Imayoshi *et al*, 2013) with *Dnmt3a* ^(fl/fl)^ mice (Kaneda *et al*, 2004) and *Dnmt3b* ^(fl/fl)^ mice (Dodge *et al*, 2005) (Fig 2A). In this mouse model, administration of tamoxifen during adulthood would lead to gene knock‐out in adult NSPCs and their differentiated progeny, but importantly, would not affect brain development.

To characterize the knock‐out efficiency of the *Nestin*::Cre‐ERT2/*Dnmt3a* ^(fl/fl)^/*Dnmt3b* ^(fl/fl)^ mouse line and to investigate the role of *de novo* DNA methylation for *in vitro* adult hippocampal NPC differentiation, we generated NPC cultures from the dentate gyrus of wildtype (WT) and knock‐out (KO) mice after tamoxifen administration (Fig 2B). Although previous studies had analyzed neuronal differentiation from *Dnmt3a*‐deficient ESCs (Wu *et al*, 2010, 2012; Ziller *et al*, 2018), the cellular consequences of deleting *de novo* DNA methyltransferases in adult hippocampal NPCs were unknown. To obtain cultures with homozygous KO of *Dnmt3a* and *Dnmt3b*, we exploited the ability of single NSPCs to grow as neurospheres (Walker *et al*, 2016) and expanded individual neurospheres to obtain clonal NPC monolayer cultures (Zocher & Kempermann, 2021). Genotyping of these cultures by polymerase chain reaction (Fig 2C) revealed that on average 82.35% ± 4.79 of activatable (neurosphere‐forming) NSPCs in KO mice contained homozygous deletions of both *Dnmt3a* and *Dnmt3b* (mean ± standard error of the mean; *n* = 5 mice). No difference in proliferation was observed between WT and KO cultures (Fig 2D; Appendix Fig S2A and B), indicating that *de novo* DNA methyltransferases are dispensable for *in vitro* adult hippocampal NPC proliferation.

Hippocampal NPCs can be *in vitro* differentiated into neurons and astrocytes by withdrawal of epidermal growth factor and fibroblast growth factor from the culture medium—a process which is associated with cell death. Two days after growth factor withdrawal, KO cultures showed reduced numbers of dead cells compared to WT cultures (Fig 2E; Appendix Fig S2C). Accordingly, we detected increased numbers of both neurons and astrocytes in differentiated KO cultures (Fig 2F and G), suggesting that deletion of *Dnmt3a*/*b* promoted survival during *in vitro* NPC differentiation. KO cultures showed higher percentages of neurons at the expense of astrocytes compared to WT cultures (Appendix Fig S2D). Moreover, neurons from KO cultures showed clear morphological differences compared to WT neurons (Fig 2H), which we confirmed by Sholl analysis (Fig 2I; Appendix Fig S2E and F). While WT neurons developed 2–4 elongated processes, KO neurons exhibited 8–12 processes with shorter dendritic length. Differentiated astrocytes also exhibited morphological differences between genotypes, with KO astrocytes covering a larger surface area than WT astrocytes (Fig 2J and K).

Together, these results showed that *de novo* DNA methyltransferases control numbers and morphology of the neuronal and astrocytic progeny of adult hippocampal NPCs *in vitro*.

### *De novo* DNA methyltransferases establish neuronal DNA methylation patterns

**Figure 3:**
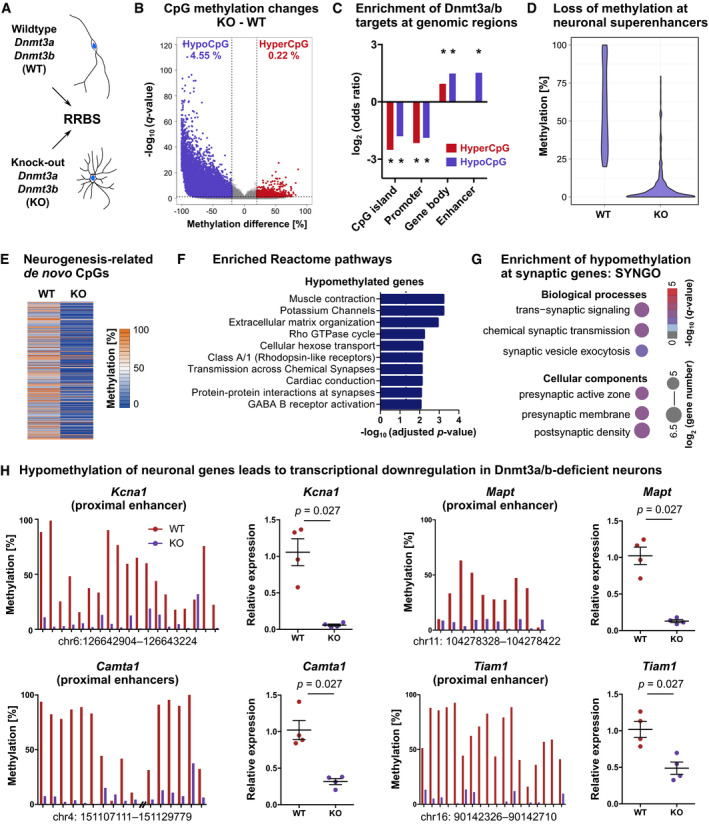
*De novo* DNA methyltransferases establish neuronal DNA methylation patterns and facilitate neuronal gene expression NPCs carrying *Dnmt3a*/*b*‐KO or WT alleles were differentiated into neurons. Neurons were isolated by FACS and their DNA methylation patterns analyzed using RRBS (*n* = 3 cell lines per genotype).We identified 22,150 CpGs that differed in methylation (*q* < 0.05 and absolute methylation difference > 20%) between KO and WT neurons, accounting to 4.55% of all CpGs covered by RRBS being hypomethylated (hypoCpG; violet) and 0.22% being hypermethylated (hyperCpG; red) in KO neurons.Hyper‐ and hypomethylated CpGs were depleted from CpG islands and promoters, but enriched at gene bodies. Hypomethylated but not hypermethylated CpGs were enriched at enhancer regions. Asterisks (*) indicate genomic regions with FDR adjusted *P* < 0.001 (*P*‐values determined by linear regression).Hypomethylated CpGs were enriched at neuronal super‐enhancers (*P* < 0.001; hypergeometric test). Depicted are methylation percentages of the 615 hypomethylated CpGs located within neuronal super‐enhancers.CpGs that gained methylation during neuronal differentiation (Fig 1) were hypomethylated in KO neurons compared to WT neurons. Depicted are the 441 CpGs covered by RRBS in both datasets.Genes containing hypomethylated cytosines (6,275 genes) in KO neurons were significantly enriched in pathways related to neuronal function as annotated in the Reactome Pathway Database.Significantly enriched biological processes and cellular components from SYNGO enrichment analysis.Dnmt3a/b‐dependent loss of DNA methylation at neuronal gene candidates was associated with reduced transcription in neurons. Depicted are mean CpG methylation levels at specified genomic regions as determined by RRBS (left) and expression fold changes in KO versus WT neurons as determined by qRT–PCR (right; *n* = 4 cell lines per genotype; *P*‐values from Mann–Whitney test; depicted are data points for every sample with genotype means ± SEM).

To validate that *Dnmt3a*/*b*‐KO influenced DNA methylation patterns of the neuronal progeny, we isolated *in vitro* differentiated neurons by FACS and compared DNA methylation profiles between WT and KO neurons using RRBS (Fig 3A). We identified focal hypomethylation at 21,109 CpGs in KO compared to WT neurons (Fig 3B; Dataset EV1), identifying those CpGs as targets of Dnmt3a and/or Dnmt3b. Hypomethylated CpGs in KO neurons were significantly enriched at gene bodies and enhancers compared to all CpGs covered by RRBS (Fig 3C). In total, Dnmt3a/b‐dependent hypomethylation targeted 1,554 putative enhancers (as defined in Zerbino *et al*, 2015) and 154 neuronal super‐enhancers (as annotated in Chen *et al*, 2020; Fig 3D). Most hypomethylated enhancers (75.74%) and super‐enhancers (98.05%) were located within gene bodies, compared to only 24.26% of hypomethylated enhancers and 1.95% of super‐enhancers that were found at distal intergenic regions. This indicates that *de novo* DNA methyltransferases mediate *de novo* DNA methylation of intragenic enhancers during neurogenesis.

In addition to hypomethylated CpGs, we found 1,041 CpGs that gained methylation in *Dnmt3a*/*b*‐KO neurons (4.70% of all differentially methylated CpGs). Those CpGs were enriched at gene bodies but not at enhancers (Fig 3C). Isolated hypermethylation after deletion of DNA methyltransferases has been reported before (Charlton *et al*, 2020) and likely represents an indirect consequence of deleting *de novo* DNA methyltransferase activity through their interaction with other molecular players.

**Figure EV2:**
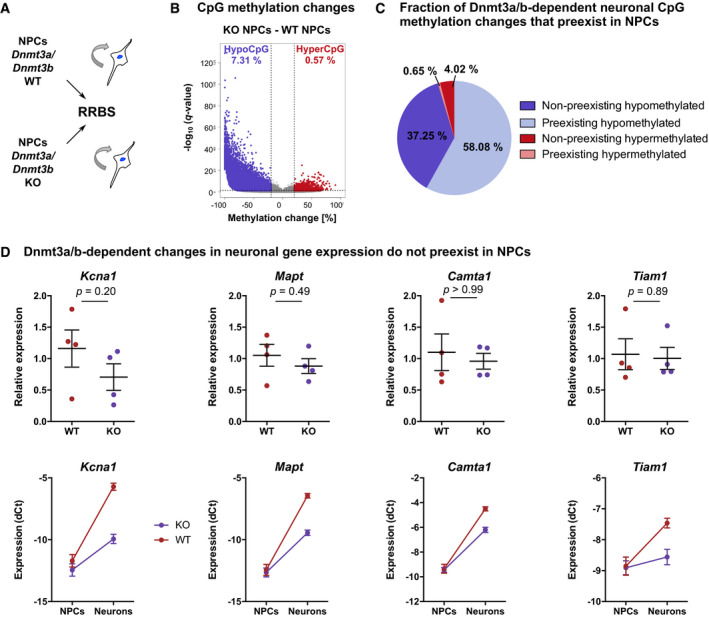
Dnmt3a/b‐dependent CpG methylation and transcriptional changes during differentiation of adult hippocampal NPCs into neurons RRBS was performed on *in vitro* cultures of proliferating NPCs that were derived from the hippocampus of adult *Dnmt3a*/*b*‐WT and KO mice (*n* = 3 cell lines per genotype).Significantly differentially methylated CpGs between KO and WT NPCs (*q* < 0.05; absolute methylation differences greater than 20%) are highlighted in violet (hypomethylated CpGs—hypoCpG) and red (hypermethylated CpGs—hyperCpG). Percentages of differentially methylated CpGs among all CpGs covered by RRBS are indicated with the respective color.Dnmt3a/b‐dependent methylation changes in neurons (see Fig 3B) separated into CpGs that were differentially methylated already in NPCs (preexistent) compared to CpG methylation differences that emerged during the course of neuronal differentiation (non‐preexistent). CpGs were further divided into hypomethylated or hypermethylated sites in KO versus WT neurons.Dnmt3a/b mediate the transcriptional up‐regulation of neuronal genes during neuronal differentiation. Depicted are expression fold changes in KO NPCs versus WT NPCs (top) and normalized expressions (normalized to *Actb*) in NPCs and neurons (bottom). Depicted *P*‐values are from Mann–Whitney test (*n* = 4 cell lines per genotype). Depicted are data points for every culture with genotype means ± SEM (top) or genotype means ± SEM (bottom).

To further dissect Dnmt3a/b‐dependent DNA methylation during neuronal differentiation, we performed RRBS on *in vitro* NPCs and compared CpG methylation patterns between WT and KO NPCs (Fig EV2A and B). We found that 60.9% of the hypomethylated CpGs in KO neurons preexisted in NPCs (Fig EV2C). Preexisting hypomethylation in KO NPCs could be explained by a differential adaptation of WT and KO NSPCs to the cell culture conditions or by a potential contribution of Dnmt3a/b to maintenance methylation during proliferation *in vitro*, that is, by compensating propagation errors of Dnmt1 similarly as reported during passaging of ESCs (Liao *et al*, 2015). Conversely, 39.1% of the hypomethylated CpGs in KO neurons did not preexist in NPCs but specifically developed during the course of neuronal differentiation (Fig EV2C). CpGs that gained methylation during neuronal differentiation (Fig 1) significantly overlapped with hypomethylated CpGs in KO neurons (hypergeometric test: *P* < 0.001) and showed a general loss of methylation in KO compared to WT neurons (Fig 3E). This confirmed that neurogenesis‐associated *de novo* methylated CpGs were targets of the canonical *de novo* DNA methyltransferases Dnmt3a and/or Dnmt3b.

Deletion of Dnmt3a/b induced hypomethylation at 6,275 genes in *in vitro* differentiated neurons, which were enriched in pathways involved in neuronal function, including potassium channels, Rho GTPase cycle, and synapse‐related pathways (Fig 3F; Dataset EV2), and at Synaptic Gene Ontology (SYNGO) terms related to synaptic signaling and synaptogenesis (Fig 3G). Hypomethylated loci included enhancers of core neuronal genes, such as *Tiam1* and *Mapt*, which are both involved in neuronal growth and axon guidance (Pallas‐Bazarra *et al*, 2016; Rao *et al*, 2019), as well as the potassium channel *Kcna1*, which controls neuronal excitation, and the transcriptional activator *Camta1*, which is required for long‐term memory formation in the hippocampus (Bas‐Orth *et al*, 2016). Gene expression analysis of those candidates by qRT–PCR revealed their significant down‐regulation in KO neurons compared to WT neurons (Fig 3H), while no expression difference was observed in NPCs (Fig EV2D). These results indicated that Dnmt3a/b mediate the transcriptional activation of neuronal genes during neurogenesis.

Together, the *in vitro* characterization suggested that *de novo* DNA methyltransferases catalyze DNA methylation of neuronal genes and that their deletion results in loss of DNA methylation at multiple neuronal enhancers, altered neuronal gene expression patterns, and aberrant neuronal morphology. Importantly, the results from our *in vitro* analysis further revealed a high efficiency of tamoxifen‐induced homozygous deletion of *Dnmt3a* and *Dnmt3b* in NSPCs, making the generated mouse model suitable for *in vivo* studies of adult hippocampal neurogenesis.

### Deletion of *de novo* DNA methyltransferases in adult hippocampal NSPCs does not influence their neurogenic activity or long‐term maintenance *in vivo*

To analyze whether *de novo* DNA methylation is functionally involved in adult hippocampal neurogenesis *in vivo*, we deleted *Dnmt3a*/*b* in NSPCs by tamoxifen administration to *Nestin*::Cre‐ERT2/*Dnmt3a* ^(fl/fl)^/*Dnmt3b* ^(fl/fl)^ mice and investigated the consequences on adult hippocampal neurogenesis.

**Figure 4:**
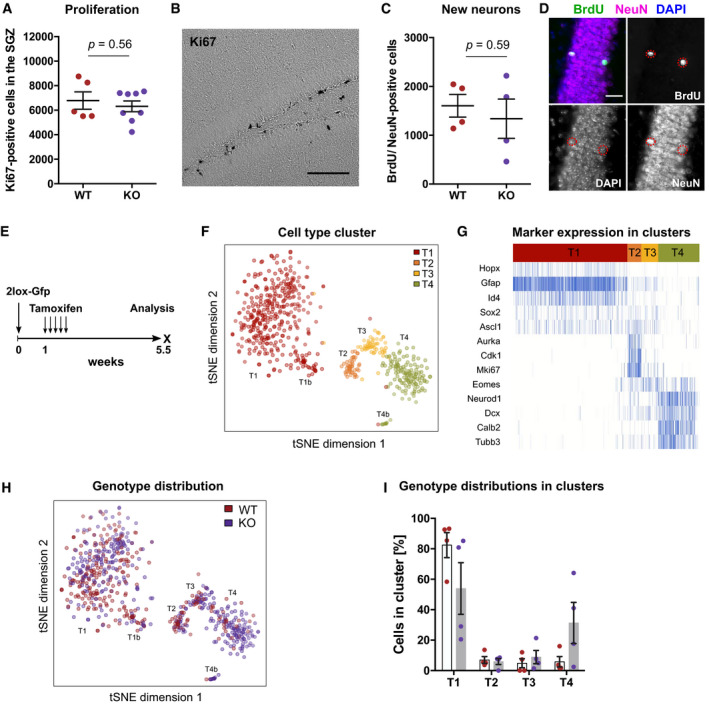
Deletion of *de novo* DNA methyltransferases does not influence neurogenic activity of adult hippocampal NSPCs *in vivo* No difference in numbers of Ki67‐positive, proliferating cells was observed between the subgranular zone (SGZ) of *Dnmt3a*/*b*‐WT and *Dnmt3a*/*b*‐KO mice 1 week after tamoxifen administration. Depicted are data points for every animal with genotype means ± SEM.Bright‐field image of Ki67‐positive cells stained using diaminobenzidine method. Scale bar: 100 µm.KO of *Dnmt3a*/*b* did not affect numbers of newborn neurons in the dentate gyrus. Mice were administered with tamoxifen and analyzed 5 weeks after the first injection. BrdU was injected 3.5 weeks before analysis. Depicted are data points for every animal with genotype means ± SEM.Fluorescence image of BrdU/NeuN‐positive newborn neurons. Scale bar: 25 µm.Experimental scheme for results depicted in Figs 4F–I, 5A and B, and EV3B and C. Recombined WT and KO cells were identified by Gfp expression in *Nestin*::Cre‐ERT2 mice.Gfp‐positive cells were isolated by FACS from WT and KO mice and their transcriptional profile analyzed using single‐cell RNA sequencing (*n* = 4 mice per genotype). *t*‐distributed stochastic neighborhood embedding (t‐SNE) clustered cells into four populations which were labeled with T1–T4 and highlighted by color. Clusters T1b and T4b were identified as sub‐clusters of T1 and T4, respectively.Marker expression identified the cell clusters as distinct cell stages during adult hippocampal neurogenesis.Distribution of WT (red) and KO cells (violet) into the different cell clusters.Percentages of WT and KO cells in the different cell clusters relative to all sufficiently covered cells (in total *n* = 293 cells from 4 mice, WT; *n* = 344 cells from four mice, KO). Depicted are percentages of cells in cluster per animal (individual data points) with genotype means ± SEM.

**Figure EV3:**
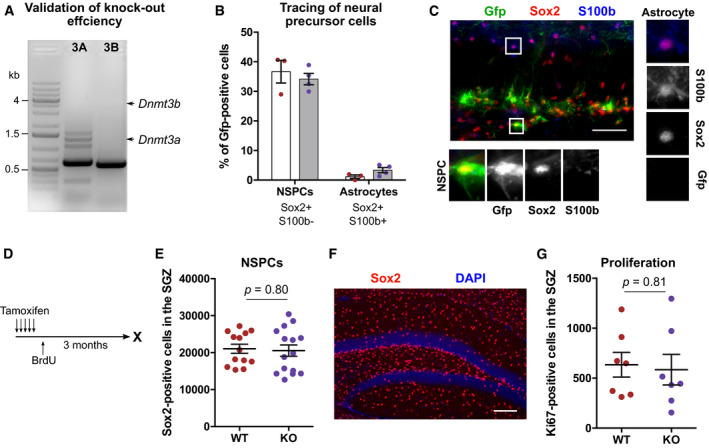
Deletion of *de novo* DNA methyltransferases does not influence long‐term maintenance of adult hippocampal NSPCs *in vivo* Validation of genomic deletion of *Dnmt3a* (3A) and *Dnmt3b* (3B) in Gfp‐positive cells by polymerase chain reaction. Arrows indicate expected sizes for wildtype *Dnmt3a* and *Dnmt3b* amplicons.No difference in the percentage of NSPCs and astrocytes among Gfp‐positive cells was detected between WT (red; *n* = 3 mice) and KO mice (violet; *n* = 4 mice). Corresponding experimental scheme is depicted in Fig 4E. Depicted are data points for every animal with genotype means ± SEM.Representative fluorescent image for detection of NSPCs and astrocytes. Depicted astrocyte is Gfp‐negative. Scale bar: 50 µm.Experimental outline for results presented in E‐G and in Fig  5D and E.No difference was found in the total numbers of NSPCs in the subgranular zone (SGZ) between WT and KO mice 3 months after tamoxifen administration (*n* = 13 mice, WT; *n* = 15 mice, KO). Depicted are data points for every animal with genotype means ± SEM (*P*‐value from unpaired *t*‐test).Representative fluorescent image for analysis of Sox2‐positive NSPCs. Scale bar: 100 µm.No difference in the total numbers of proliferating cells in the SGZ between WT and KO mice 3 months after recombination (*n* = 7 mice per genotype). Depicted are data points for every animal with genotype means ± SEM (*P*‐value from unpaired *t*‐test).

We did not observe a difference between WT and KO mice in NSPC proliferation acutely 1 week after tamoxifen administration (Fig 4A and B), nor in the total number of newborn neurons 5 weeks after induction of deletion (Fig 4C and D). To investigate a potential influence on the dynamics of adult neurogenesis, we traced NSPCs by injecting a lentivirus carrying a Cre‐dependent Gfp vector into WT and KO mice (Fig 4E). After validating the tamoxifen‐induced deletion of *Dnmt3a* and *Dnmt3b* in FAC‐sorted Gfp‐positive cells from KO mice (Fig EV3A), we analyzed cell stage distributions of Gfp‐positive cells after a chase period of 4 weeks. We did not detect a difference in the percentages of label‐retaining NSPCs or astrocytes (Fig EV3B and C). Additionally, no differences in total numbers of NSPCs or proliferating cells were found between KO and WT mice 3 months after tamoxifen administration (Fig EV3D–G). These results suggested that *de novo* DNA methyltransferases are dispensable for NSPC maintenance in the adult hippocampus.

During the process of adult neurogenesis, precursor cells progress through different developmental cell stages before they mature to hippocampal granule cells (Kempermann *et al*, 2004). To further resolve a potential role of *de novo* DNA methyltransferases in controlling NSPCs and immature precursor cell stages, we labeled recombined NSPCs using a Cre‐dependent Gfp vector as described before (Fig 4E), isolated Gfp‐positive cells from the dentate gyrus of WT and KO mice 4 weeks after tamoxifen administration and performed single‐cell RNA sequencing. We obtained sufficient sequencing reads for 293 WT cells and 344 KO cells. Visualizing the cells using *t*‐distributed stochastic neighborhood embedding (t‐SNE) revealed four distinct clusters (Fig 4F) that corresponded to the developmental cell stages of adult hippocampal neurogenesis (Fig 4G). Cluster T1 showed expression of NSPC markers but absence of proliferation markers (Fig 4G; Appendix Fig S3), identifying it as the quiescent neural stem cell population, while cluster T2 contained the proliferating progenitor cells. Cells in cluster T3 corresponded to the post‐mitotic, neuronally committed precursor cells that expressed *Eomes*. Cluster T4 contained the immature neurons with high expression of markers such as *Dcx*, *Neurod1* and *Calb2*. KO cells did not differ in their distribution into the distinct precursor cell stages compared to WT cells (Fig 4H), but exhibited trends toward higher numbers of immature neurons in cluster T4 (Fig 4I). Using unbiased differential expression analyses, we identified 444 genes that changed expression between the cell stages, but could not detect any significantly differentially expressed genes between WT and KO cells. These results indicated that deletion of *de novo* DNA methyltransferases did not cause major gene expression changes in NSPCs and early post‐mitotic cells. Together with the results from the histological characterization, the cluster analysis suggested that acute genetic deletion of *Dnmt3a*/*b* did not influence the neurogenic activity of hippocampal NSPCs.

Despite a chase period of 4 weeks before analysis, we could not identify neurons based on expression of mature neuronal markers, such as Calb1 (Appendix Fig S3) and observed an overrepresentation of precursor cell stages compared to the results from our histological analysis. This can likely be attributed to the rupture of neuronal axons and membranes during the FACS process, which prohibited analysis of Dnmt3a/b‐dependent transcriptional changes in neurons *in vivo*.

### Deletion of *de novo* DNA methyltransferases in hippocampal NSPCs impairs maturation of newborn neurons *in vivo*

**Figure 5:**
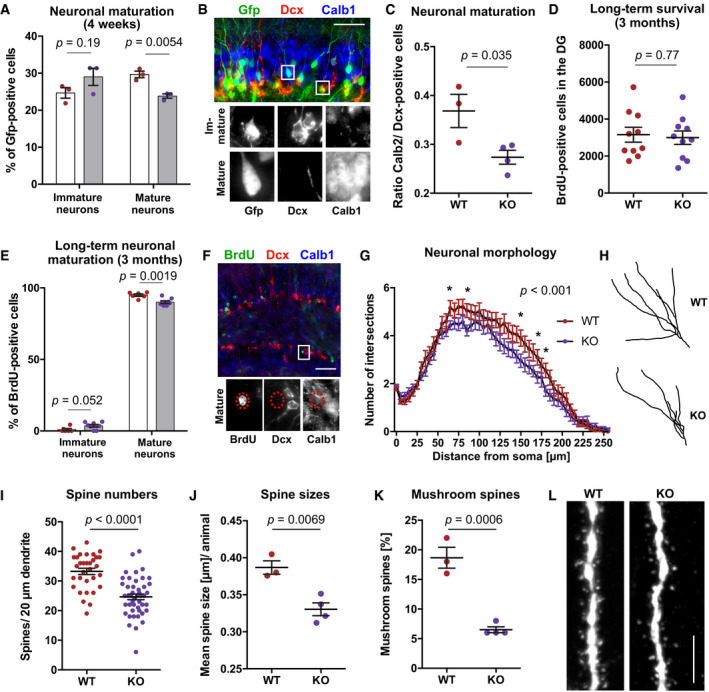
*De novo* DNA methyltransferases control maturation rate, dendritic outgrowth and spine formation of adult‐born neurons in the hippocampus *Dnmt3a*/*b*‐KO mice show reduced percentages of mature neurons (Calb1‐positive, Dcx‐negative) among Gfp‐positive cells compared to WT mice (*n* = 3 mice, WT; *n* = 3 mice, KO). Immature neurons were identified as Dcx‐positive and Calb1‐negative. Experimental scheme as shown in Fig 4E.Representative fluorescence image of mature and immature neurons. Scale bar: 50 µm.KO mice exhibit reduced total numbers of Calb2‐positive cells relative to Dcx‐positive cells in the dentate gyrus.No difference in long‐term survival of newborn cells (BrdU‐positive) was observed between WT and KO mice. BrdU was injected 3 months before analysis (experimental scheme depicted in Fig EV3D).Long‐term deficit in maturation of adult‐born neurons in KO mice. Mature neurons were identified as BrdU‐positive, Calb1‐positive, and Dcx‐negative (*n* = 6 mice, WT; *n* = 8 mice, KO).Representative fluorescence image of the immunostaining used for detection of mature neurons in Fig 5E. Scale bar: 100 µm.Sholl analysis of Gfp‐positive cells revealed reduced dendritic outgrowth of adult‐born neurons in KO mice (*n* = 20 neurons, WT; *n* = 23 neurons, KO). Depicted *P*‐value reports genotype effect from two‐way ANOVA (distance to soma: *P* < 0.001). Asterisks highlight data points with *P* < 0.05 after multiple testing correction (Holm method) of repeated *t*‐tests. Data points are means ± SEM. Experimental scheme as depicted in Fig 4E.Representative neuronal traces of WT and KO mice.Adult‐born neurons of KO mice exhibit reduced numbers of dendritic spines in the outer molecular layer (*n* = 32 neurons from three mice, WT; *n* = 46 neurons from four mice, KO). Depicted are individual data points for every neuron with genotype means ± SEM (*P*‐value from unpaired *t*‐test).Mean spine size per animal was reduced in KO mice (*n* = 50 spines per mouse).Mean percentage of mushroom spines (diameter of spine head ≥ 0.45 µm) per animal was lower in KO mice compared to WT mice.Representative fluorescence image of dendritic spines in WT and KO mice. Scale bar: 5 µm. Data information: Depicted *P*‐values in (A), (C–E), (J, K) are from unpaired *t*‐test. Graphs show data points for every animal with genotype means ± SEM. Further statistical details are reported in Appendix Table S5.

The trend toward higher percentages of immature neurons in KO mice observed in the single‐cell cluster analysis suggested a potential influence of *de novo* DNA methyltransferases on the dynamics of neuronal maturation. To substantiate this observation, we performed immunohistochemistry for immature and mature neuronal markers on WT and KO mice that had been injected with a 2lox‐Gfp construct as described earlier (Fig 4E). Four weeks after recombination, KO mice exhibited a significantly lower percentage of mature neurons (Calb1‐positive/Dcx‐negative) compared to WT mice but a trend toward increased numbers of immature neurons (Dcx‐positive/Calb1‐negative; Fig 5A and B). Similarly, when we compared total numbers of cells expressing the post‐mitotic neuronal marker Calb2 with numbers of cells expressing immature neuronal marker Dcx, we detected a significant decrease in the ratio of Calb2‐positive cells compared to Dcx‐positive cells in KO mice (Fig 5C), suggesting a delay of neuronal maturation in KO mice. To investigate long‐term consequences of *Dnmt3a*/*b*‐KO on newborn neuron maturation, we analyzed BrdU‐positive cells for expression of neuronal markers 3 months after BrdU and tamoxifen injections (Fig EV3D). KO and WT mice did not differ in total numbers of BrdU‐positive cells (Fig 5D), suggesting that *Dnmt3a*/*b*‐KO did not affect long‐term survival of adult‐born cells. While the vast majority of BrdU‐positive cells (> 85%) were identified as mature neurons (Calb1‐positive, Dcx‐negative) in both genotypes, KO mice maintained slightly lower percentages of mature neurons compared to WT mice (Fig 5E and F).

Dendritic outgrowth and spine formation are integral parts of neuronal maturation and prerequisites for their experience‐dependent activation and functional integration (Toni & Schinder, 2015). Neurons in KO mice showed a mild but significant decrease in dendritic outgrowth compared to neurons in WT mice (Fig 5G and H), which is consistent with their delayed maturation. Moreover, KO neurons exhibited significantly fewer dendritic spines in the outer molecular layer (Fig 5I). Spines in KO mice had smaller head diameters (Fig 5J) and comprised of a lower percentage of mushroom spines (Fig 5K and L). Together these results demonstrated that *de novo* DNA methyltransferases control maturation dynamics, dendritic outgrowth, and spine formation of newborn neurons in the hippocampus.

### Neurogenesis‐associated *de novo* DNA methylation is not required for the pro‐neurogenic effect of environmental enrichment

**Figure 6:**
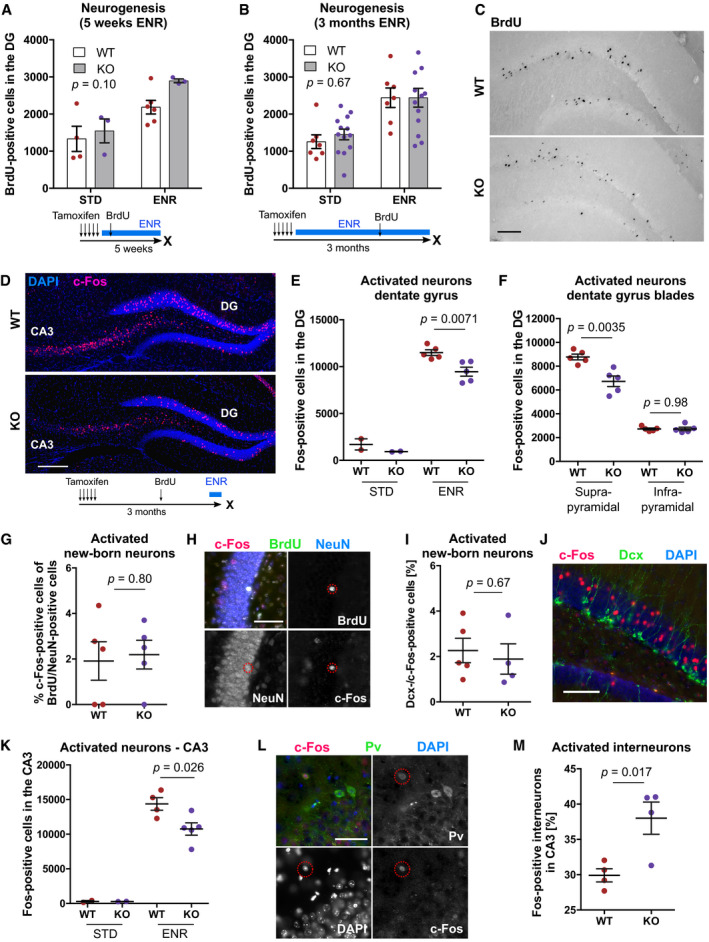
Deletion of *de novo* DNA methyltransferases does not abolish the pro‐neurogenic effect of environmental enrichment but modifies hippocampal excitability A, BQuantification of adult‐born (BrdU‐positive) cells in the dentate gyrus (DG) of *Dnmt3a*/*b*‐KO and WT mice after 5 weeks or 3 months of environmental enrichment (ENR) or standard housing (STD). BrdU was injected 4.5 weeks before analysis. Depicted *P*‐values correspond to genotype effects from two‐way ANOVA. Influences of ENR were significant with *P* < 0.01. Depicted are data points for every animal with group means ± SEM.CBright‐field images of BrdU‐positive cells stained using diaminobenzidine method from WT and KO mice housed in ENR for 3 months. Scale bar: 100 µm.DFluorescence images of c‐Fos‐positive cells in the DG and CA3 of WT and KO mice. Scale bar: 200 µm.ENumbers of c‐Fos‐positive cells in the DG without (STD) and with acute stimulation in ENR for 1 h (*n* = 2, WT STD; *n* = 2, KO STD; *n* = 5, WT ENR; *n* = 5, KO ENR).FNumbers of c‐Fos‐positive cells in suprapyramidal and infrapyramidal blades of the DG after acute stimulation in ENR (*n* = 5 mice, WT; *n* = 5 mice, KO).GPercentage of newborn neurons (BrdU/NeuN‐positive) that expressed *c‐Fos* after ENR (*n* = 5 mice, WT; *n* = 5 mice, KO).HRepresentative fluorescent image of *c‐Fos* expression in BrdU/NeuN‐positive cells in the DG. Scale bar: 25 µm.IPercentage of c‐Fos‐positive cells that expressed *Dcx* in WT and KO mice (*n* = 5 mice, WT; *n* = 4 mice, KO).JRepresentative fluorescent image of c‐Fos‐positive cells and Dcx‐positive cells in the DG. Scale bar: 100 µm.KReduced numbers of c‐Fos‐positive cells in CA3 of KO mice (*n* = 4 mice, WT; *n* = 5 mice, KO).LFluorescent image of c‐Fos expression in Parvalbumin (Pv)‐positive interneurons in CA3. Scale bar: 50 µm.MA higher percentage of Pv‐positive interneurons in CA3 expressed *c‐Fos* in KO mice (*n* = 4 mice, WT; *n* = 4 mice, KO). Data information: Depicted *P‐*values in panels (E–M) are from unpaired *t*‐test. Graphs show data points for every animal with group means ± SEM. Further statistical details are presented in Appendix Table S5.

Environmental enrichment (ENR) is a strong pro‐neurogenic stimulus that predominantly increases the survival of newborn neurons (Kempermann *et al*, 1997; Körholz *et al*, 2018). We have previously shown that ENR changed DNA methylation patterns at neuronal plasticity genes in the dentate gyrus (Zocher *et al*, 2021), which prompted us to analyze whether cell‐intrinsic DNA methylation changes in NSPCs and immature neurons are required for the pro‐neurogenic effect of ENR. To analyze whether ENR‐induced *de novo* DNA methylation is functionally involved in newborn neuron survival, we administered tamoxifen to WT and KO mice and housed them in ENR or STD for 5 weeks or 3 months. Deletion of *de novo* DNA methyltransferases did not abolish the ENR‐induced increase in the numbers of newborn cells in the hippocampus (Fig 6A–C), suggesting that cell‐intrinsic *de novo* DNA methylation during neuron formation is not required for the pro‐neurogenic effect of ENR.

### Neurogenesis‐associated *de novo* DNA methylation modulates experience‐dependent activation in the hippocampal circuitry

Adult‐born neurons control neuronal activity in the hippocampal circuitry (Ikrar *et al*, 2013; Berdugo‐Vega *et al*, 2020). Specifically, recent studies suggested that immature, newborn neurons contribute to sparse activation of the hippocampus by activating inhibitory interneurons (Drew *et al*, 2016) as well as through their monosynaptic inhibition on mature granule cells (Luna *et al*, 2019). To explore whether Dnmt3a/b‐deficient neurons are impaired in their functional integration, we acutely exposed mice to ENR for 1 h and quantified the numbers of activated, c‐Fos‐positive neurons. KO mice showed significantly fewer c‐Fos‐positive cells in the dentate gyrus after stimulation in ENR compared to WT mice (Fig 6D and E). This reduction in neuronal activation was specific to the suprapyramidal blade of the dentate gyrus (Fig 6F), which is consistent with the previously reported role of adult‐born neurons in specifically inhibiting this blade in response to novelty exposure (Luna *et al*, 2019). No difference was detected in the percentage of BrdU/NeuN‐positive cells that expressed *c‐Fos* (Fig 6G and H), nor in the percentage of c‐Fos‐positive cells that expressed *Dcx* (Fig 6I and J). This suggested that not reduced cell‐intrinsic activation of adult‐born KO neurons but their inhibitory input onto mature granule cells might explain the observed reduction in c‐Fos‐positive cells in the dentate gyrus. Consistent with the known feedforward inhibition of dentate granule cells to pyramidal cells in CA3 (Tuncdemir *et al*, 2019), we found reduced total numbers of c‐Fos‐positive cells also in area CA3 (Fig 6K). Additionally, a significantly higher percentage of Parvalbumin‐positive interneurons in CA3 expressed *c‐Fos* in KO mice (Fig 6L and M), likely contributing to increased feedback and feedforward inhibition in dentate gyrus and CA3, respectively. Since immature neurons are hyper‐excitable and known to activate CA3 interneurons (Toni & Schinder, 2015), the observed delay in newborn neuron maturation could explain the increased interneuron activation in *Dnmt3a*/*b*‐KO mice. Together, these results indicate that deletion of *de novo* DNA methyltransferases during adult neurogenesis modulates neuronal activation in the hippocampal circuitry in response to novel environment exposure.

### Neurogenesis‐associated *de novo* DNA methylation is crucial for hippocampus‐dependent learning and memory

**Figure EV4:**
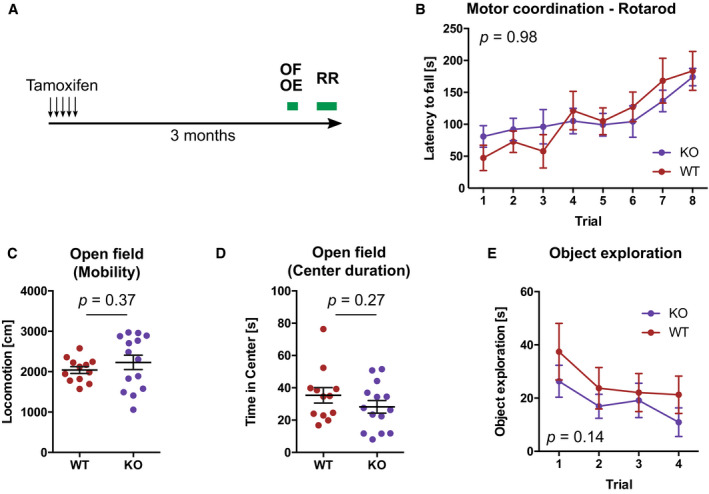
Deletion of *Dnmt3a* and *Dnmt3b* in adult NSPCs and their progeny does not influence motor coordination or exploratory activity Three months after administration of tamoxifen, mice were tested in one trial of open field test (OF) followed by four trails of object exploration test (OE). One week later, mice were tested on the rotarod (RR).Rotarod performance in WT and KO mice. Depicted *P*‐value corresponds to genotype effect from non‐parametric longitudinal model (*n* = 7, WT; *n* = 11, KO). Depicted are means ± SEM.Distance mice moved in the open field (*n* = 12, WT; *n* = 14, KO; *P*‐value from Mann–Whitney test). Depicted are data points per animal with genotype means ± SEM.Time mice spent in the center of the open field (*n* = 12, WT; *n* = 14, KO; *P*‐value from Mann–Whitney test). Depicted are data points per animal with genotype means ± SEM.Time mice spent around objects. In trial 4, one object was replaced with a novel object. Depicted *P*‐value corresponds to genotype effect from non‐parametric longitudinal model (*n* = 7, WT; *n* = 11, KO). Depicted are means ± SEM.

**Figure 7:**
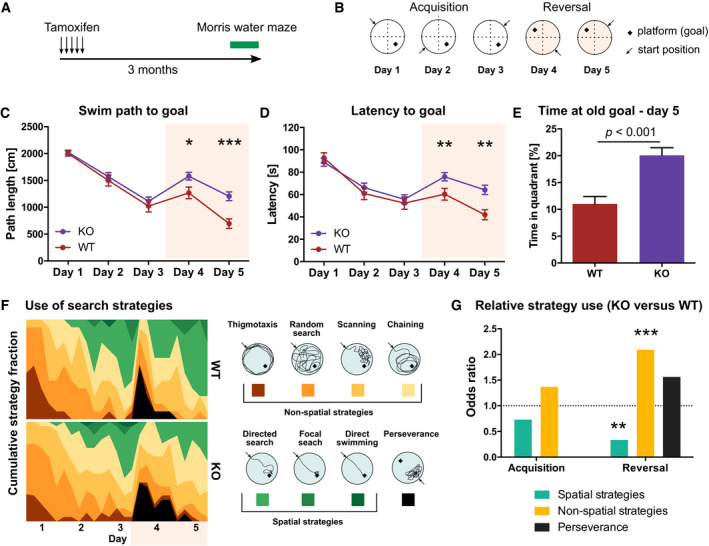
Blocking *de novo* DNA methylation during adult hippocampal neurogenesis impairs hippocampus‐dependent learning and memory Mice with adult NSPC‐specific deletion of *Dnmt3a* and *Dnmt3b* (KO) and wildtype littermates (WT) were tested in the Morris water maze 3 months after administration of tamoxifen (*n* = 13 mice, WT; *n* = 23 mice, KO).Protocol for the Morris water maze test.Average distance animals swam per day to find the hidden platform.Average time animals needed to reach the platform per day. Note that KO mice showed increased path length and latency on days 4 and 5 compared to WT mice.KO mice spent on day 5 more time in the quadrant in which the goal was located during the acquisition phase compared to WT mice.Heatmaps depicting the percentage of individual search strategies used by WT and KO mice per trial (left). Color scheme for the classification into spatial and non‐spatial search strategies (right).Odds ratio of strategy use in KO compared to WT mice separated by acquisition and reversal phase (*P*‐values were determined by linear regression). Data information: *P < 0.05; **P < 0.01; ***P < 0.001. Panels (C–E) show means ± SEM. Unpaired Mann–Whitney test was used to determine significance in (C–E).

Adult‐born neurons in the hippocampus contribute to cognitive flexibility which can be assessed using behavioral tests measuring learning and memory. To analyze whether the observed changes in neuronal maturation and hippocampal activation lead to cognitive deficits in KO mice, we tested animals in different behavioral tests. WT and KO mice showed no differences in motor coordination on the rotarod (Fig EV4A and B), exploration of an open field arena (Fig EV4C and D) or exploration of novel objects (Fig EV4E), but a specific impairment in reversal learning in the Morris water maze (Fig 7). Previous studies have demonstrated that adult‐born hippocampal neurons contribute to the cognitive flexibility required for re‐learning of the platform position after reversal of its initial location in the Morris water maze (Garthe *et al*, 2009).

No difference was detected between WT and KO mice in their ability to learn the initial platform position during the acquisition phase (Fig 7A–D). However, after reversal of the platform position, KO mice swam significantly longer distances (swim path; Fig 7C) and needed more time to find the hidden platform compared to WT mice (Fig 7D). Additionally, on the second day after reversal, KO mice spent more time in the quadrant in which the platform was located before reversal (Fig 7E). These results suggested that KO mice are less flexible in re‐learning the new platform position after reversal. As an additional adult neurogenesis‐dependent measure of learning, we analyzed the navigational strategies mice used to allocate the hidden platform. While there was no difference in search strategy choice between KO and WT mice during the acquisition phase, KO mice used significantly fewer hippocampus‐dependent “spatial” strategies and more “non‐spatial” strategies after reversal (Fig 7F and G). Together, our results indicate that *de novo* DNA methylation during adult neurogenesis is required for proper maturation and functional integration of adult‐born neurons into the hippocampus.

## Discussion

Here, we present a comprehensive dissection of the function of *de novo* DNA methyltransferases during adult hippocampal neurogenesis. We found that *de novo* DNA methyltransferases target enhancers and gene bodies of neuronal genes during adult neurogenesis to establish neuron‐specific methylomes and neuronal gene expression patterns. Our mechanistic analyses demonstrated a critical role of neurogenesis‐associated *de novo* DNA methylation specifically for the maturation of newborn neurons, for hippocampal excitability and for the cognitive enhancement conveyed by adult hippocampal neurogenesis. Together, our study provides new insights into the role of *de novo* DNA methylation for an adult stem cell system that influences life‐long behavior and cognition of individuals.

Using RRBS, we here identified focal *de novo* DNA methylation of adult neurogenesis‐related genes during the *in vitro* differentiation of adult hippocampal NPCs into neurons. Pronounced *de novo* DNA methylation was also reported during the *in vitro* neuronal differentiation from cortical progenitor cells of the embryonic brain (Luo *et al*, 2019), during differentiation of ESCs into motor neurons (Ziller *et al*, 2018) or pyramidal neurons (Mohn *et al*, 2008) and during the reprogramming of fibroblasts into neurons (Luo *et al*, 2019). Other studies found increased 5‐hydroxymethylcytosine at neurogenic genes during embryonic neurogenesis (Hahn *et al*, 2013; Noack *et al*, 2019). Since RRBS does not distinguish between 5‐methylcytosine and 5‐hydroxymethylcytosine, it is currently unknown how much of the here detected *de novo* DNA methylation is attributed to 5‐hydroxymethylcytosine. Moreover, RRBS does not provide genome‐wide coverage of CpGs, and hence, global DNA methylation changes during adult neurogenesis remain unknown. Furthermore, while RRBS yields information for a high percentage of CpG islands, it provides reduced coverage of enhancers. Since enhancers are known to exhibit cell type‐specific DNA methylation patterns (Xie *et al*, 2013; Mo *et al*, 2015), our study potentially underestimated the percentage of CpGs that change methylation, and DNA methylation changes outside of the covered areas remain unidentified. Performing whole‐genome bisulfite sequencing and high‐resolution RNA sequencing in the different cell stages of the neurogenic trajectory *in vivo* could help in future studies to identify their genome‐wide relationship and to present an exhaustive list of genes that might control adult hippocampal neurogenesis by changing their methylation patterns. Meanwhile, our study showed that deletion of *Dnmt3a*/*b* induced hypomethylation of the genes *Mapt*, *Kcna1*, *Camta*, and *Tiam1* in neurons and blocked their up‐regulation during neuronal differentiation. These results are in accordance with the detected enrichment of *de novo* DNA methylation at neurogenesis‐related genes, including *Mapt*, and consistent with findings from Wu and colleagues who showed that Dnmt3a‐dependent DNA methylation of neuronal genes activates transcription through repression of polycomb proteins (Wu *et al*, 2010). Moreover, many neuronal transcription factors specifically bind to methylated genomic sites such as Pax6, Nfatc4, Npas2, Tfcp2, and Egr4 (Yin *et al*, 2017). Our DNA methylation data will be an important starting point for future experiments to understand how *de novo* DNA methyltransferases are recruited to neuronal genes and how they interact with other molecular players to control adult hippocampal neurogenesis.

Our study suggests that neurogenic commitment is epigenetically pre‐determined in adult NSPCs and independent of further *de novo* DNA methylation during neuronal differentiation. Previous studies showed that differentiation of ESCs into NSPCs is associated with pronounced *de novo* DNA methylation (Mohn *et al*, 2008; Ziller *et al*, 2018). Blocking these changes through deletion of *Dnmt3a*/*b* in ESCs significantly impaired the terminal differentiation of NSPCs into neurons, resulting in increased neuronal cell death and reduced percentages of neuronal progeny but enhanced astrogliogenesis (Wu *et al*, 2012; Ziller *et al*, 2018). Similarly, mice with global knock‐outs of *Dnmt3a* showed reduced postnatal neurogenesis in the hippocampus and subventricular zone (Wu *et al*, 2010). In our study, deleting *Dnmt3a* and *Dnmt3b* specifically in adult NSPCs did not impair the *in vivo* production of new neurons at the level of NSPC proliferation, neuronal fate specification, or survival. This suggested that *de novo* DNA methylation associated with the terminal differentiation of NSPCs into neurons is not required for making a neuron *per se*. Neuronal fate determination might be dependent rather on the maintenance of the previously established DNA methylation patterns in NSPCs than on continuous activity of *de novo* DNA methyltransferases during terminal differentiation. Consistent with this model, inducible deletion of *Dnmt1* in adult NSPCs strongly reduced numbers of newborn neurons in the hippocampus, while it left proliferation or long‐term maintenance of NSPCs unaffected (Noguchi *et al*, 2015).

Our study demonstrated that Dnmt3a/b control neuronal maturation both during neuronal differentiation *in vitro* and adult hippocampal neurogenesis *in vivo*. However, the phenotypic consequences of *Dnmt3a*/*b*‐KO seemed to be more pronounced *in vitro*, with stronger morphological alterations as well as a pro‐survival effect on neurons and astrocytes in KO cultures which was absent *in vivo*. This discrepancy in phenotypes could be explained by differences in extracellular environments and/or a higher extent of preexisting DNA methylation differences in NPCs *in vitro* compared to NSPCs *in vivo*. Neuronal maturation and survival are highly dependent on extracellular input, such as signaling molecules (e.g., neurotransmitters, ions, metabolites) as well as contacts to extracellular matrix and neighboring cells (Vicidomini *et al*, 2020), which differ between *in vivo* and *in vitro* conditions, and can influence DNA methylation patterns (Guo *et al*, 2011). Moreover, the detected hypomethylation of KO NPCs *in vitro* is likely much less pronounced in NSPCs *in vivo*, if present at all. Since Dnmt3a/b compensate for imperfect maintenance by Dnmt1, continued passaging of NPCs could have amplified differences in maintenance methylation *in vitro*, similarly to what has been reported for passaging of *Dnmt3a*/*b*‐deficient ESCs (Liao *et al*, 2015). In contrast, activated NSPCs *in vivo* undergo only 2–4 cell divisions before they differentiate into neurons (Pilz *et al*, 2018; Bottes *et al*, 2021), likely resulting in reduced effects of *Dnmt3a*/*b*‐KO on maintenance methylation *in vivo*. Furthermore, also NSPCs respond to extracellular input (Pötzsch *et al*, 2021), and since the *Dnmt3a*/*b*‐KO was induced in NSPCs *in vivo*, transfer to cell culture conditions might have triggered *de novo* CpG methylation in WT NPCs but not in *Dnmt3a*/*b*‐KO NPCs. Together, these findings highlight how crucial *in vivo* studies are for understanding processes which are as dependent on environmental input as adult hippocampal neurogenesis.

Rather than controlling mere numbers of newborn neurons in the hippocampus, *de novo* DNA methyltransferases were crucial for the morphological and functional maturation of the new neurons. Evidence of whether this deficit is a direct consequence of hampered *de novo* DNA methylation during neurogenesis or whether mature neurons need continuous Dnmt3a/b‐dependent epigenetic remodeling would require additional cell stage‐specific deletion of Dnmt3a/b during adult neurogenesis. However, the observed long‐term reduction in percentages of mature neurons at the expense of immature neurons and the resulting Dnmt3a/b‐dependent changes in hippocampal excitation after novel environment exposure are suggestive of a direct role of *de novo* DNA methyltransferases in facilitating neuronal maturation. Additional support comes from knock‐outs of Mecp2—the essential reader of *de novo* methylation in neurons—which revealed a specific requirement of Mecp2 for spine formation and the transition from immature to mature neuronal stages (Smrt *et al*, 2007). The hypomethylation of genomic loci with known roles in synapse organization and the reduced numbers of spines observed in Dnmt3a/b‐deficient neurons could suggest alterations in pre‐ and/or postsynaptic connections as a potential mechanism underlying the cognitive deficits of Dnmt3a/b‐KO mice. Understanding how Dnmt3a/b‐deficient adult‐born neurons change hippocampal excitability—whether through altered input sensitivity, quality of their firing pattern, altered neuronal connectivity, or a combination of other mechanisms—could help, in future experiments, to unravel the mechanisms of how adult‐born neurons promote cognitive flexibility.

Proper control of neuronal DNA methylation patterns is necessary for life‐long brain function (Oliveira *et al*, 2012; Gontier *et al*, 2018), and its dysregulation is a proposed mechanism underlying several neurological diseases, including Alzheimer's disease and depression (Han *et al*, 2018; Li *et al*, 2019). Here, we show that *de novo* DNA methyltransferases are required for the contribution of adult neurogenesis to hippocampus‐dependent learning and memory. Our study provides evidence for a role of *de novo* DNA methylation in facilitating neuronal maturation in the adult brain and a resource for further mechanistic studies toward understanding the molecular control of healthy brain function.

## Materials and Methods

### Animals

Female C57BL/6JRj mice were purchased from Janvier at an age of 5 weeks. *Nestin*::EGFP mice (Yamaguchi *et al*, 2000), *Dcx*::GFP mice (Gong *et al*, 2003), and *Nestin*::Cre‐ERT2/*Dnmt3a* ^(fl/fl)^/*Dnmt3b* ^(fl/fl)^ mice were bred and maintained at the animal facility of the Center for Regenerative Therapies Dresden. To generate *Nestin*::Cre‐ERT2/*Dnmt3a* ^(fl/fl)^/*Dnmt3b* ^(fl/fl)^ mice, Dnmt3a‐2lox (Kaneda *et al*, 2004) and Dnmt3b‐2lox (Dodge *et al*, 2005; both obtained from RIKEN, Japan) were crossed and the offspring mated with *Nestin*::Cre‐ERT2 mice (Imayoshi *et al*, 2006). Experimental animals were obtained by backcrossing *Nestin*::Cre‐ERT2^(+/−)^/*Dnmt3a* ^(fl/fl)^/*Dnmt3b* ^(fl/fl)^ mice with *Nestin*::Cre‐ERT2^(−/−)^/*Dnmt3a* ^(fl/fl)^/*Dnmt3b* ^(fl/fl)^ mice. WT animals (*Nestin*::Cre‐ERT2^(−/−)^/*Dnmt3a* ^(fl/fl)^/*Dnmt3b* ^(fl/fl)^) were the siblings of the KO animals that did not contain the *Nestin*::Cre‐ERT2 allele. For lineage tracing experiments, WT animals were *Nestin*::Cre‐ERT2^(+/−)^/*Dnmt3a* ^(wt/wt)^/*Dnmt3b* ^(wt/wt)^ and derived from the offspring of the original cross of *Dnmt3a* ^(fl/wt)^‐*Dnmt3b* ^(fl/wt)^ with *Nestin*::Cre‐ERT2. Parental strains were maintained on C57BL/6 backgrounds.

Mice were housed in standard polycarbonate cages (Type II, Tecniplast) and maintained on a 12‐h light/dark cycle at the animal facility of the Center for Regenerative Therapies Dresden and the German Center for Neurodegenerative Diseases. Food and water were provided *ad libitum*. All experiments were conducted in accordance with the applicable European and National regulations (Tierschutzgesetz) and were approved by the local authority (Landesdirektion Sachsen: file numbers 25‐5131/354/63 and 25‐5131/365/9).

### Administration of drugs

Bromodeoxyuridine (BrdU; Sigma #B5002) was dissolved in 0.9% sodium chloride (to 10 mM) and injected intraperitoneally at a final concentration of 50 mg/kg body weight. Tamoxifen (Sigma #T5648‐5G) was dissolved in corn oil (Sigma) to 50 mg/ml and was administered at 250 mg/kg body weight via oral gavage (one injection every day for five consecutive days).

### Fluorescence‐activated cell sorting

Micro‐dissected dentate gyri were cut into pieces with a scalpel blade and enzymatically dissociated using the Neural Tissue Dissociation Kit P (Miltenyi Biotec) according to the manufacturer's manual. Dissociated cells were washed with Hank's balanced salt solution (HBSS; Gibco) and separated using a 40‐µm cell strainer (Falcon, BD Bioscience). To remove dead cells, propidium iodide (1 µg/ml; Thermo Fisher Scientific) was added directly before the sort. For labeling of neurons, cells were incubated with NeuroFluor™ NeuO (1:500; Stem Cell Technologies) for 15 min at 4°C in HBSS. FACS was performed using a BD FACSARIA™ III sorter and the software FACSDiva v 8.0.1 (both BD Bioscience). Data were analyzed using FlowJo v 10 (Tree Star, Inc.).

### Reduced representation bisulfite sequencing

Genomic DNA was isolated using the QIAamp DNA Micro Kit (QIAGEN). RRBS libraries were prepared from 100 ng genomic DNA using the Premium RRBS Kit (Diagenode) and purified twice using Agencourt AMPure XP beads (Beckman Coulter; 1X bead volume). Quality and concentration of RRBS libraries were determined using the High Sensitivity NGS Fragment Analysis Kit (Advanced Analytical) and a fragment analyzer with capillary size of 33 cm (Advanced Analytical). Sequencing was performed using a NextSeq500 platform in a 75 bp single end mode with a minimum sequencing depth of 10 million reads per sample. Numbers of sequencing reads and coverage of all samples are depicted in Appendix Table S1.

### Quantitative polymerase chain reaction

Total RNA was isolated using the RNeasy Micro Kit (QIAGEN). RNA was reversely transcribed into complementary DNA using SuperScript™ II Reverse Transcriptase with Oligo(dT)_12–18_ primers and 1 μl dNTPs (10 mM each; all Thermo Fisher Scientific). Quantitative real‐time polymerase chain reactions were performed using the QuantiFast SYBR Green PCR Kit (QIAGEN) and the CFX ConnectTM Real‐Time PCR Detection System (Bio‐Rad). Primers for quantitative PCRs are listed in Appendix Table S2.

### Single‐cell RNA sequencing

Single cells were collected into 384‐well plates pre‐loaded with 2.3 µl lysis buffer (50 mM guanidine hydrochloride, 17.4 mM dNTPs, 2.2 µM SMART dT30VN primer). Cells from WT and KO mice were randomized across three plates. The plates were sealed and stored at −80°C. Smart‐Seq2 libraries were generated using Tecan Freedom EVO and Nanodrop II (Bionex) systems as previously described (Picelli *et al*, 2013).

In brief, cells were incubated at 95°C for 3 min. Reverse transcription was performed by adding 2.7 µl reverse transcription mix (SuperScript II buffer (Invitrogen), 9.3 mM DTT, 370 mM Betaine, 15 mM MgCl_2_, 9.3 U SuperScript II RT (Invitrogen), 1.85 U recombinant RNase Inhibitor (Takara), 1.85 µM template‐switching oligo) to each lysed cell using a Nanodrop II liquid handling system (BioNex) followed by incubation at 42°C for 90 min and inactivation at 70°C for 15 min. Amplification of cDNA was performed for 16 cycles with 7.5 µl preamplification mix containing KAPA HiFi HotStart ReadyMix and 2 µM ISPCR primers. cDNA was purified with 1X Agencourt AMPure XP beads (Beckman Coulter) and eluted in 14 µl nuclease‐free water. Concentration and cDNA size was analyzed for selected representative wells using a High Sensitivity D5000 assay for the Tapestation 4200 system (Agilent). cDNA was diluted to an average of 200 pg/µl and 100 pg cDNA from each cell was tagmented using Nextera XT DNA Library Preparation Kit (Illumina). The tagmentation reaction was incubated at 55°C for 8 min before removing Tn5 from the DNA by adding 0.5 µl NT buffer per well. 1 µl well‐specific indexing primer mix from Nextera XT Index Kit v2 Sets A‐D and 1.5 µl NPM was added to each well and the tagmented cDNA was amplified for 14 cycles according to manufacturer's specifications. PCR products from all wells were pooled and purified with 1X Agencourt AMPure XP beads (Beckman Coulter). The fragment size distribution was determined using a High Sensitivity D5000 assay for the Tapestation 4200 system (Agilent), and library concentration was determined using a Qubit dsDNA HS assay (Thermo Fischer). Libraries were clustered at 1.4 pM concentration using High Output v2 chemistry and sequenced on a NextSeq500 system SR 75bp with 2*8bp index reads.

### Culture and analysis of *in vitro* neural precursor cells

Micro‐dissected dentate gyri were enzymatically dissociated using the Neural Tissue Dissociation Kit P (Miltenyi Biotec). Cells were separated using a 40‐µm strainer, plated at approximate single‐cell dilution in 96‐well plates in NPC medium supplemented with 20 mg/ml Heparin (MP Biomedicals) and incubated for 14 days. NPC medium consisted of Neurobasal medium (Gibco™) supplemented with 1× B‐27 supplement (Gibco™), 1× GlutaMAX™ supplement (Gibco™), 10,000 U/ml Pen/Strep (Gibco™), 20 mg/ml human FGF2 (PeproTech) and 20 mg/ml human EGF (PeproTech). For generation of clonal lines, single neurospheres were transferred into 96‐well plates, mechanically disrupted using a 200‐µm pipet tip and expanded as adherent monolayer cultures. Cells were maintained on dishes coated with poly‐d‐lysine (Sigma‐Aldrich) and laminin (Roche Diagnostics). NPCs were passaged at a confluence of 80% using Accutase (Gibco™). Differentiation was induced by withdrawing human EGF and FGF from the NPC medium for 5 days.

For genotyping of cell lines, genomic DNA was isolated using the QIAamp DNA Micro Kit (QIAGEN) and gene fragments were amplified using DreamTaq DNA Polymerases (Thermo Fisher Scientific). For calculation of knock‐out efficiency, a minimum of 10 cell lines from each of the five animals were expanded and genotyped. Five cell lines with homozygous knock‐outs of *Dnmt3a* and *Dnmt3b* were randomly selected for characterization.

For proliferation assays, cells were incubated with 10 µM EdU for 3 h and analyzed using Click‐iT^TM^ EdU Alexa Fluor 647 Flow Cytometry Assay Kit (Thermo Fisher Scientific). Cell death was investigated using FITC Annexin V/Dead Cell Apoptosis Kit (Thermo Fisher Scientific). Cells were recorded with a BD™ LSRII (BD Biosciences) and analyzed using FlowJo v10™.

For analysis of cell differentiation, cells were cultured on poly‐d‐lysine/laminin‐coated glass coverslips and fixed with 4% paraformaldehyde for 10 min. Cells were washed in PBS and blocked for 90 min with PBS supplemented with 10% normal donkey serum (Jackson ImmunoResearch) and 0.2% Triton X‐100 (Carl Roth). Primary antibodies were incubated for 1 h and secondary antibodies for 30 min in PBS supplemented with 3% normal donkey serum and 0.2% Triton X‐100. Nuclei were stained with Hoechst 33342 (1:4,000 in PBS) for 10 min. All incubation steps were performed at room temperature. Coverslips were mounted onto glass slides using Aquamount (Polysciences Inc.).

Differentiated cells were imaged using an Apotome Fluorescence Miscroscope (Zeiss) with a 20× objective. Cells were analyzed on at least 10 image fields per sample. Image fields were randomly selected on the coverslips. Cell quantifications were performed using Fiji v1.0. Statistical details are presented in Appendix Table S5.

### Preparation and administration of lentiviruses

Lentiviruses were produced by polyethyleneimine co‐transfection of 293T cells with the transfer vector encoding for HIV‐1 gag/pol and VSV‐G as previously described (Artegiani *et al*, 2012; Berdugo‐Vega *et al*, 2020). The transfer vector was designed such that removal of the floxed‐stop codon after tamoxifen‐dependent recombination allowed for the expression of the GFP reporter specifically in the NSC progeny. One day after transfection, cells were switched to serum‐free medium, and 1 day later, the supernatant was filtered and centrifuged at 55,000 *g* for 4 h. The viral particles were resuspended in 40 μl of PBS per 10 cm petri dish and further concentrated using centrifugal filters (Amicon) yielding ca. 40 μl of virus suspension per construct with a titer of 10^8^–10^9^ IU/ml as assessed on HEK cells. Viral particles (1 μl) were stereotaxically injected in the DG hilus of isoflurane‐anesthetized mice as previously reported (Artegiani *et al*, 2012) using a nanoliter‐2000 injector (World Precision Instruments) and a stereotaxic frame Model 900 (Kopf Instruments) at ± 1.6 mm mediolateral, −1.9 anteriorposterior, and −1.9 mm dorsoventral from bregma with a constant flow of 200 nl/min.

### Immunohistochemistry

Mice were anesthetized by intraperitoneal injection of a mixture of ketamine (100 mg/kg bodyweight; WDT) and xylazine (10 mg/kg bodyweight; Serumwerk Bernburg AG) diluted in 0.9% sodium chloride. Mice were transcardially perfused with 0.9% sodium chloride. Brains were removed from the skull, bisected, and post‐fixed in 4% paraformaldehyde prepared in phosphate buffer (pH 7.4) overnight at 4°C. For cryoprotection, brains were transferred into 30% sucrose in phosphate buffer until they sank down before they were cut into 40‐ or 60‐µm‐thick coronal sections using a dry‐ice‐cooled copper block on a sliding microtome (Leica, SM2000R). Sections were stored at −20°C in cryoprotectant solution (25% ethyleneglycol, 25% glycerol in 0.1 M phosphate buffer, pH 7.4).

For immunofluorescent stainings, free‐floating sections were washed in phosphate‐buffered saline (PBS) and unspecific antibody binding sites were blocked by incubating sections in PBS supplemented with 10% donkey serum (Jackson ImmunoResearch) and 0.2% Triton X‐100 (Carl Roth) for 90 min at room temperature. Primary antibodies were applied overnight at 4°C and secondary antibodies for 2 h at room temperature. Antibodies were diluted as depicted in Appendix Tables S3 and S4 in PBS supplemented with 3% donkey serum and 0.2% Triton X‐100. Nuclei were labeled with DAPI (1:4,000; Jackson ImmunoResearch). Sections were mounted onto glass slides and cover‐slipped using Aquamount (Polysciences Inc.). Fluorescence stainings were imaged using a Zeiss Apotome equipped with an AxioCam MRm camera and the software AxioVision 4.8 (Zeiss).

BrdU‐labeled cells or Ki67‐labeled cells were stained using peroxidase method as previously described (Körholz *et al*, 2018). Briefly, sections were incubated in 0.6% hydrogen peroxide for 30 min to inhibit endogenous peroxidase activity. For BrdU stainings, sections were incubated in pre‐warmed 2.5 M hydrochloric acid for 30 min at 37°C. Blocking and antibody incubation was performed as described for immunofluorescent stainings. For detection, Vectastain ABC‐Elite reagent (9 µg/ml of each component; Vector Laboratories LINARIS) was used with diaminobenzidine (0.075 mg/ml; Sigma) and 0.04% nickel chloride. All washing steps were performed in Tris‐buffered saline. Mounted sections were cleared with Neo‐Clear and cover‐slipped using Neo‐Mount (both Millipore). BrdU‐positive cells were counted using a bright‐field microscope (Leica DM 750).

All representative images for immunohistochemistry presented in the paper were taken from WT mice unless stated otherwise.

### Cell quantifications for immunohistochemistry

Total cell numbers were quantified on every sixth section along the entire rostro‐caudal axis of the dentate gyrus (9–11 sections per animal). No separation into dorsal and ventral hippocampus was performed. For all experiments, cells were quantified on both blades of the dentate gyrus, with the exception of the experiment presented in Fig 6F, for which cells were quantified separately in suprapyramidal and infrapyramidal blades. Phenotyping of BrdU‐/Gfp‐/c‐Fos‐ or Pv‐positive cells was performed on every sixth section along the entire rostro‐caudal axis of the hippocampus. A minimum of 60 cells per animal was phenotyped for every analysis. Statistical details for all quantifications are presented in Appendix Table S5.

### Analysis of dendrites and spines

Dendrites and spines were analyzed on every sixth section along the entire rostro‐caudal axis of the hippocampus. Imaging was performed using a Confocal LSM 780 NLO and the software ZEN 2011 SP7. Dendritic lengths in the hippocampus were analyzed on 60‐µm‐thick sections with a 20× objective and 1.7‐µm stack intervals. Sholl analysis was performed in Fiji v1.0 using the plugin Simple Neurite Tracer with a radius step size of 5 µm. Spines were quantified on images taken from 40‐µm‐thick sections with a 63× glycerol objective, using 0.8‐µm stack intervals and 1.8× zoom. For spine analysis, dendrites were randomly selected independent of dendritic order.

### Animal behavior

#### Environmental enrichment

The enriched environment was designed such that it provided sensory, physical, social, and cognitive stimulation to the mice. The enclosure was made of white polycarbonate walls and covered an area of 0.74 m^2^. For cognitive stimulation, the cage was equipped with tunnels, plastic toys, and hide‐outs, which were rearranged twice per week. To increase social complexity, at least 10 mice were housed at the same time in one enclosure. Dirty bedding material and toys were replaced once per week. Control mice stayed in standard polycarbonate cages (Type II, Tecniplast).

#### Rotarod

Mice were placed on an Economex Rotarod (Columbus Instruments) and trained on three consecutive days with three trials per day. The rotating cylinder started with a speed of 4 rpm and accelerated by 0.1 rpm. Time until mice fell off was measured.

#### Open field and novel object exploration test

Open field and novel object exploration tests were performed as previously described (Körholz *et al*, 2018) with few modifications. Briefly, mice were placed in a square arena (50 cm × 50 cm) and their movements were recorded for five minutes using Ethovision XT (Noldus). For the novel object recognition test, two identical objects were placed in the arena and the time mice spent around the objects was analyzed. On the fourth day of testing, one of the objects was replaced with a novel object. Objects and novel object location were randomized among mice.

#### Morris water maze

Mice were tested in the reference memory version of the Morris water maze test as previously described (Garthe *et al*, 2009). Briefly, a pool with a diameter of 2 m was filled with opaque colored water that was equilibrated to room temperature (20–22°C). A quadratic platform with a surface of 10 cm^2^ was placed in the pool hidden under the water surface. Mice were tested on five consecutive days with five trials per day and an inter‐trial interval of 1–2 h. In every trial, mice were allowed to search for the hidden platform for 120 s and were placed on the platform afterward for 15 s irrespective of trial outcome. Starting positions changed every day, but remained constant for all trials on 1 day. After the third testing day, the platform position was changed to the opposite quadrant. Swim paths and latencies were recorded using a camera (Logitech) and the software EthoVision XT (Noldus). Search strategies were manually scored from swim tracks with the experimenter blinded to the groups. Differences in strategy use were analyzed by fitting a general linear model with binomial distribution using the R function glm. Odds ratios are the exponential coefficients of the model.

### Bioinformatic data analysis

#### DNA methylation data

Fastq reads were trimmed using Trim Galore 0.4.4 and the function *Cutadapt* 1.8.1 in RRBS mode and mapped against mm10 using Bismark 0.19.0 (Krueger & Andrews, 2011). Detection of differentially methylated cytosines was performed using methylKit v1.12.0 (Akalin *et al*, 2012). Briefly, methylation levels were extracted from sorted Binary Alignment Map files using the function *processBismarkAln*. Data were filtered for cytosines with a minimum coverage of 10 reads and a maximum coverage of 99.9% percentile in all samples using the functions *filterByCoverage* and *unite*. Differentially methylated cytosines were identified using the *methDiff* function applying the chi‐squared test, basic overdispersion correction, and multiple testing correction using the sliding linear model (SLIM) method, a significance threshold of *q* < 0.05 and a threshold for absolute cytosine methylation differences > 20%.

Genomic locations of CpG islands, enhancers, exons, and introns were retrieved from the UCSC Genome Browser (Karolchik *et al*, 2004). Predicted enhancer regions were retrieved from the Ensembl Regulatory Build of the corresponding UCSC Genome Browser track (Zerbino *et al*, 2015). Super‐enhancers were downloaded from SEA: Super‐Enhancer Archive (Chen *et al*, 2020). Neuronal super‐enhancers used for analysis correspond to mouse super‐enhancers derived from cortex and cerebellum. Promoters were defined as 500 bp upstream and downstream of transcription start sites. Promoter regions were excluded from gene bodies. Overlaps of cytosines weres performed using the function *subsetByOverlaps* of the R package Genomic Ranges v1.38.0 (Lawrence *et al*, 2013). Enrichment at genomic regions was determined by calculating odds ratios using logistic regression followed by multiple testing correction of *P*‐values using FDR method.

Differentially methylated CpGs were annotated to the gene with the nearest transcription start site using data tables downloaded from Ensembl BioMart (download as of 28.04.2020; Zerbino *et al*, 2018). For pathway analysis, we used the R package ReactomePA (Yu & He, 2016) with the following parameters: *P*valueCutoff = 0.05, maxGSSize = 200, minGSSIZE = 10. SYNGO enrichment was performed using the online tool at https://www.syngoportal.org/ with default parameters (Koopmans *et al*, 2019). MANGO enrichment analysis was performed using the 359 annotated genes with published functional role in adult neurogenesis (Overall *et al*, 2012). All enrichment analyses were performed with differentially methylated genes as query lists and all genes covered by RRBS as background lists. For the background list, genes were considered covered by sequencing if at least one CpG was sequenced with a minimum of 10 reads in all samples.

#### Single‐cell RNA sequencing data

Single‐cell data were demultiplexed using bcl2fastq2 v2.20 and aligned to the mouse reference transcriptome mm10 using kallisto v0.44.0 with default parameters. Transcript levels were quantified as transcripts per million reads (TPM). TPM counts were imported into R and transcript information was summarized on gene levels. We removed genes which did not have more than 10 reads for more than 500 genes. We further applied an upper threshold of 10% mitochondrial gene expression to filter out extreme values (potentially arising from damaged cells). We detected an average of 71,914.12 ± 3,149.22 (mean ± SEM) reads per cell and 2,824 ± 42.13 (mean ± SEM) genes per cell. The expression of mitochondrial genes as a fraction of the total transcriptome was in the range of 0.12–9.9% with a mean of 3.8 ± 0.088% (mean ± SEM). The pagoda2 package (Fan *et al*, 2016) was used for variance normalization (gam.k = 10), identification of cell clusters (PCA: nPcs = 100, n.odgenes = 3e3; *k*‐nearest neighbor: *k* = 50, perplexity = 30, min.group.size = 10) and *t‐*distributed stochastic neighbor embedding (*t*‐SNE). Clusters were annotated based on marker genes, and two smaller clusters containing contaminating microglia and oligodendrocytes were removed and the clustering recalculated to yield six clusters covering the neurogenic trajectory. Log‐transformed normalized gene expression values were then exported from pagoda2 for further analysis in R. Differential expression analysis was performed using edgeR (McCarthy *et al*, 2012) on the untransformed count data which had been adjusted using the zinbwave package (Risso *et al*, 2018).

### Statistics

Statistics was preformed using R v3.6.3 or GraphPad Prism v6. All experiments were performed with the experimenter being blinded to the experimental groups. We applied unpaired *t*‐tests (two‐sided), two‐way ANOVA, Mann–Whitney tests, or non‐parametric longitudinal models as indicated in the figure legends. Adjustment for multiple comparisons was performed as appropriate (specified in the figure legends). All plots with sample sizes *n* < 20 show individual data points.

## Author contributions

SZ conceptualized the study, performed the experiments, analyzed the data and wrote the manuscript. FC, GB‐V, and GK reviewed and edited the manuscript. RWO analyzed single‐cell RNA sequencing and curated data. GB‐V prepared viruses and performed stereotactic injections. NR and VSA performed FACS. AK coordinated breeding of mice. CS performed genotyping of mice and cell lines. NR and SS helped with immunohistochemistry. KH and JLS performed single‐cell RNA sequencing. FC supervised virus injections. GK supervised the study and acquired funding.

## Conflict of interest

The authors declare that they have no conflict of interest.

## Supporting information



Review Process File

Appendix

Expanded View Figures PDF

Dataset EV1

Dataset EV2

## Data Availability

Datasets produced in this study are available at GEO (accession number GSE167955; http://www.ncbi.nlm.nih.gov/geo/query/acc.cgi?acc=GSE167955).
