# Supplementary material for: De novo DNA methylation controls neuronal maturation during adult hippocampal neurogenesis
Source: EMBO J. 2021 Aug 2;40(18):e107100. doi: 10.15252/embj.2020107100 (PMC8441477; doi:10.15252/embj.2020107100)
Supplement: Supplementary file 2 — Appendix [file EMBJ-40-e107100-s004.pdf]

Appendix

Table of content:

Appendix Figures S1-3

Appendix Tables S1-5

Appendix Figures

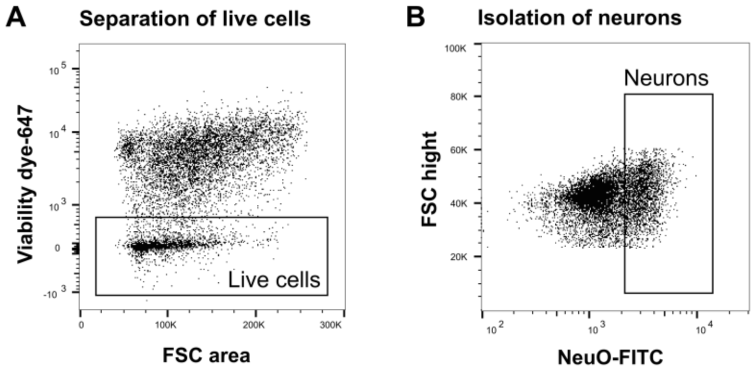

Appendix Figure S1: Isolation of *in vitro* differentiated neurons.

A, After separation of single cells based on their forward scatter (FSC) and side scatter (SSC) properties, dead cells were removed using a viability dye.

B, Neurons were identified by fluorescence intensity of the neuronal dye NeuroFluor (NeuO) in live cells.

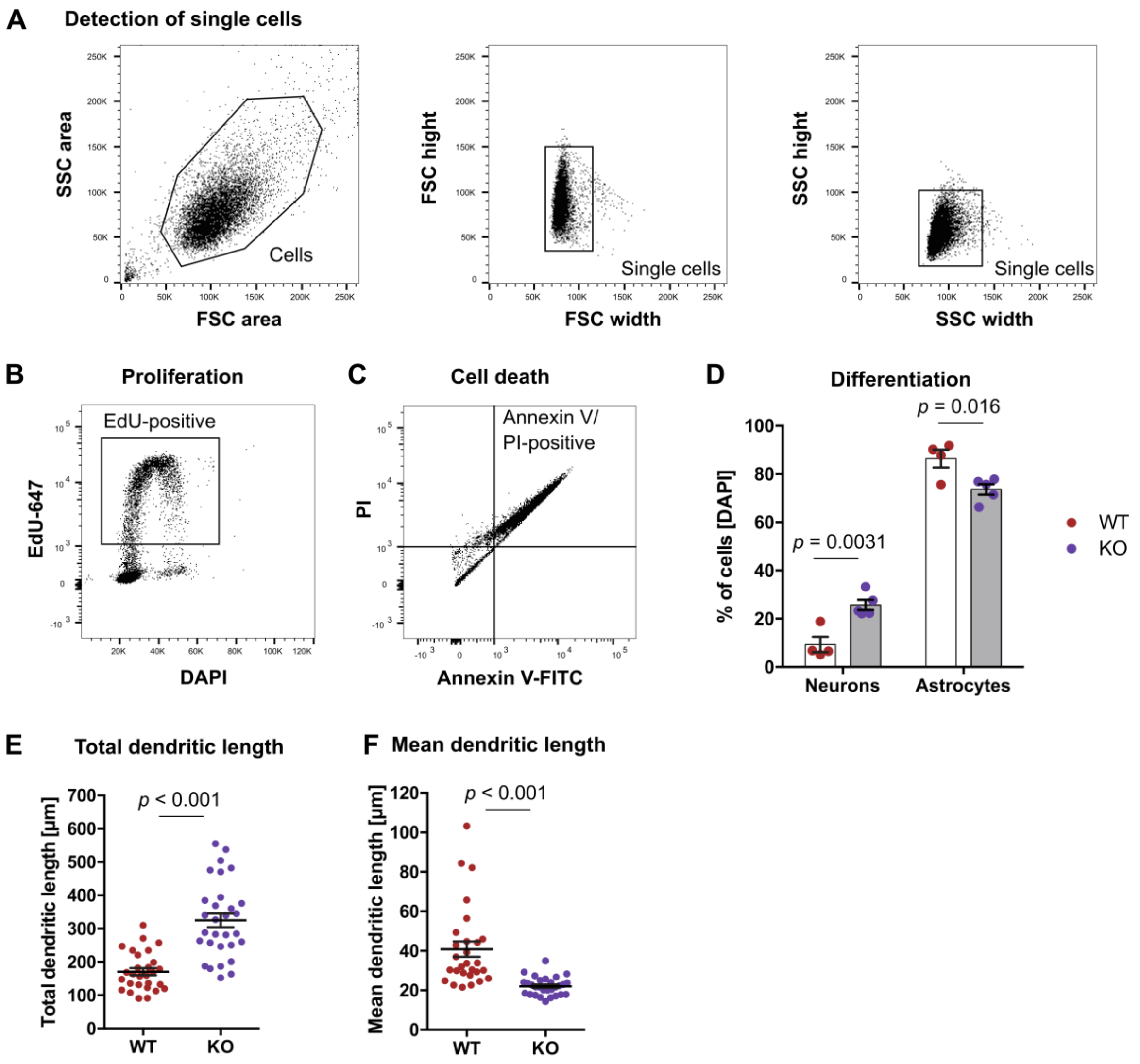

Appendix Figure S2: Analysis of *in vitro* NPCs and differentiated neurons.

A, FACS strategy used for detection of neural precursor cells (NPCs). Single cells were identified by plotting forward scatter (FSC) and side scatter (SSC).

B, Cells in S-phase were identified based on EdU fluorescence intensity (corresponds to Fig. 2D).

C, Detection of dead cells by intensity of apoptosis marker Annexin V and incorporation of propidium iodide (PI; corresponds to Fig. 2E).

D, Differentiated *Dnmt3a/b*-KO cultures contained increased percentages of Tubb3-positive neurons and reduced percentages of GFAP-positive astrocytes compared to WT cultures. Depicted *p*-values from unpaired *t*-test.

E-F, Tubb3-positive neurons in differentiated KO cultures exhibited elevated total dendritic length but reduced mean dendritic length compared to WT cultures, suggesting that KO

neurons grew a larger number of shorter dendrites. Depicted *p*-values in E-F are from unpaired *t*-test.

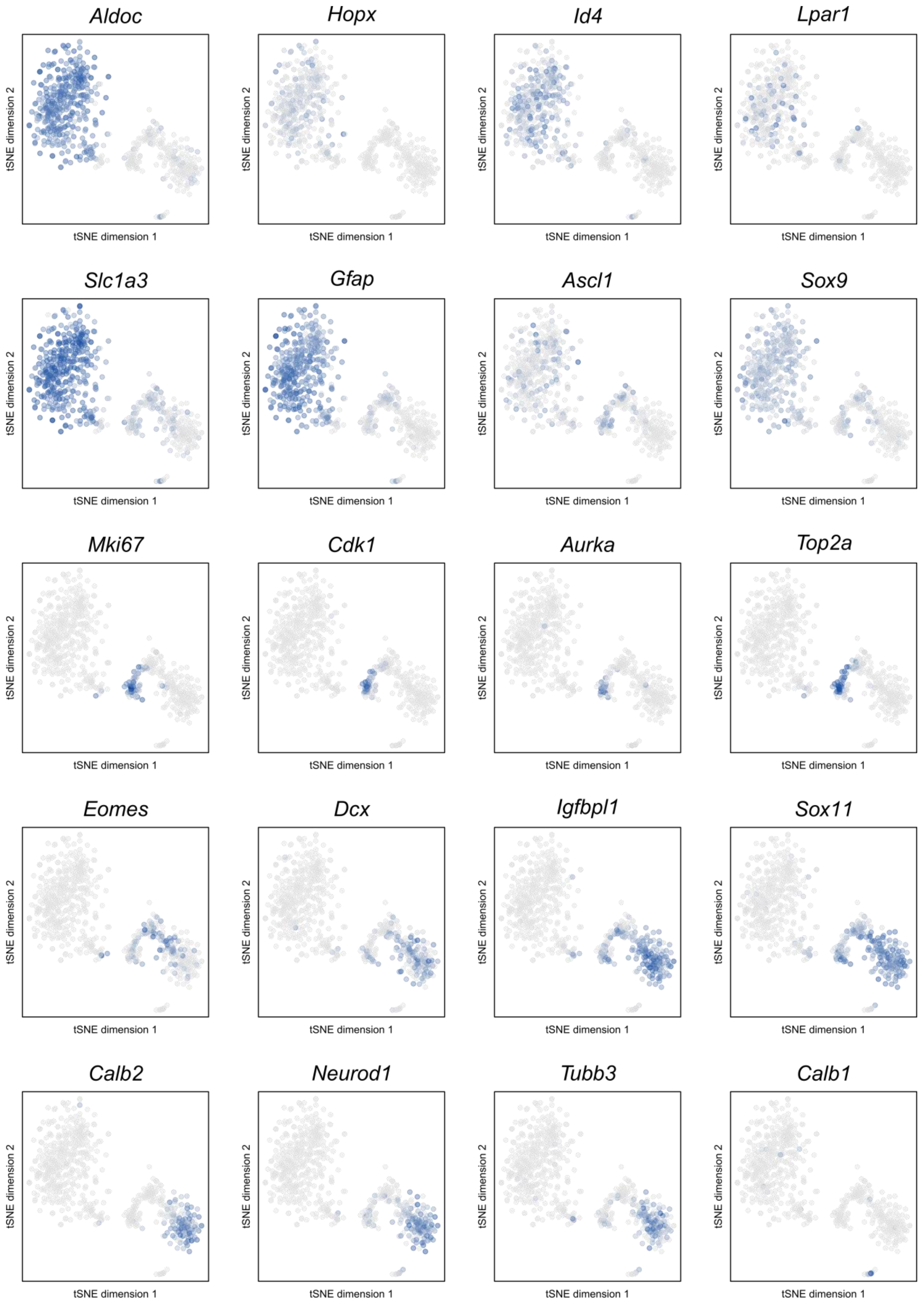

Appendix Figure S3: Marker gene expression in cell clusters. Depicted are t-SNE plots for marker genes that label specific cell stages during adult hippocampal neurogenesis.

Appendix Table S1: Sample information of the RRBS data

Sample	Sample description	Sequencing reads	Covered cytosines (≥ 10x)	Covered CpGs (≥ 10x)	Mean sequencing depth (coverage)/ CpG	Figures
NPC1	Dnmt3a/b WT	15236174	8371398	998060	15.55	1; EV2
NPC1	Dnmt3a/b WT	19677495	9224287	1198780	18.16	1; EV2
NPC3	Dnmt3a/b WT	16605749	8623578	1059404	16.61	1; EV2
Neuron1	Dnmt3a/b WT	13855307	7197657	1190660	21.36	1; 3
Neuron2	Dnmt3a/b WT	10412335	6189769	918599	16.78	1; 3
Neuron3	Dnmt3a/b WT	13934717	6807307	1205035	23.45	1; 3
NPC4	Dnmt3a/b KO	16274656	8800300	1075483	16.19	EV2
NPC5	Dnmt3a/b KO	11724813	7182666	767015	13.72	EV2
NPC6	Dnmt3a/b KO	10973854	6900260	662652	13.28	EV2
Neuron4	Dnmt3a/b KO	13535640	7701026	1001149	17.36	3
Neuron5	Dnmt3a/b KO	15660040	8338379	1176847	18.95	3
Neuron6	Dnmt3a/b KO	15431985	8277766	1146612	18.42	3

Appendix Table S2: List of primer sequences.

**Primer name with target gene**	**Primer sequence (5‘-3‘)**	**Annealing temperature**	**Used for**
Act_q_fw<br>Act_q_re	ACCCGCGAGCACAGCTTC<br>ACATGCCGGAGCCGTTGTC	59 °C	qRT-PCR,<br>RT-PCR
Dnmt3a_q_fw<br>Dnmt3a_q_rev	AGGTTTGATCCAAGCAGGTG<br>ACTTGACTGGTGCCGAGAGT	59 °C	RT-PCR
Dnmt3b_q_fw<br>Dnmt3b_q_rev	CATGTGGCTAGTCCTCACGA<br>GGGAATGGATTTCCCTAAGC	59 °C	RT-PCR
Dnmt3a_fw<br>Dnmt3a_rev	CTACATTGCCTCCGAGGTGT<br>GGCCACCACATTCTCAAAGA	62°C	Genotyping
Dnmt3b_fw<br>Dnmt3b_rev	CGCAGGAAAGATTGGAACAT<br>GTGAGCAGCAGACACCTTGA	62°C	Genotyping
Camta1_q_fw<br>Camta1_q_rev	GCAGTACGATGAGCTGGCTG<br>AGCTGGCATTAGAAGACGGC	59 °C	qRT-PCR
Mapt_q_fw<br>Mapt_q_rev	CGCCAGGAGTTTGACACAATG<br>GTTCCTCCGCTCCATCATCG	59 °C	qRT-PCR
Kcna1_q_fw<br>Kcna1_q_rev	GCCATTGTGTCGGTCATGGT<br>TGTTGTCGATGCGGTGGATG	59 °C	qRT-PCR
Tiam1_q_fw<br>Tiam1_q_rev	CACTGTCTTTCCGAGGGTGC<br>TCGATCCCACTGTCCGCAAA	59 °C	qRT-PCR

Appendix Table S3: List of primary antibodies.

**Antibody target**	**Antibody description**	**Manufacturer**	**Catalog number**	**Dilution**
BrdU	Monoclonal rat	AbD Serotec	-	1:500
Calbindin (Calb1)	Monoclonal mouse	Swant	300	1:500
Calretinin (Calb2)	Polyclonal goat	Swant	CG1	1:500
c-Fos	Polyclonal rabbit	Synaptic Systems	226003	1:1000
Dcx	Polyclonal goat	Santa Cruz	sc-8066	1:500
GFAP	Polyclonal rabbit	DakoCytomation	Z0334	1:1000
Gfp	Chicken	Abcam	ab13970	1:750
Ki67	Polyclonal rat	eBioscience	14-5698-82	1:750
NeuN	Polyclonal rabbit	Abcam	104225	1:250
NeuN	Monoclonal mouse	Chemicon	MAB377	1:250
Pv	Monoclonal mouse	Swant	Pv235	1:1000
S100b	Polyclonal rabbit	Abcam	ab52642	1:500
Sox2	Polyclonal goat	R&D	AF2018	1:500
Tubb3a	Monoclonal mouse	Promega	G712A	1:1000

Appendix Table S4: List of secondary antibodies.

**Antibody**	**Manufacturer**	**Catalog number**	**Dilution**	**Combined with**
Alexa Fluor 488 Donkey Anti-Rat IgG	Jackson ImmunoResearch	712-545-153	1:1000	Anti-BrdU
Biotin Donkey Anti-Rat IgG	Jackson ImmunoResearch	712-065-153	1:1000	Anti-BrdU, Anti-Ki67
Cy3 Donkey Anti-Goat IgG	Jackson ImmunoResearch	705-165-151	1:1000	Anti-Dcx, Anti-Sox2, Anti-Calb2
Cy3 Donkey Anti-Mouse IgG	Polyclonal rabbit	715-165-151	1:2000	Anti-Tubb3
Alexa Fluor 647 Donkey Anti-Mouse IgG	Jackson ImmunoResearch	715-605-151	1:1000	Anti-NeuN, Anti-Calb1
Alexa Fluor 488 Donkey Anti-Rabbit IgG	Jackson ImmunoResearch	711-545-152	1:2000	Anti-GFAP
Cy3 Donkey Anti-Rabbit IgG	Jackson ImmunoResearch	711-165-152	1:1000	Anti-c-Fos
Alexa Fluor 647 Donkey Anti-Rabbit IgG	Jackson ImmunoResearch	711-605-152	1:1000	Anti-S100b, Anti-NeuN
Alexa Fluor 488 Donkey Anti-Chicken IgY	Jackson ImmunoResearch	703-545-155	1:1000	Anti-Gfp

Appendix Table S5: Statistics for cell quantifications

**Figure**	**Phenotype**	**Number of replicates WT**	**Number of replicates KO**	***P*-value from non-parametric test (Mann-Whitney)**
2D	% EdU-positive cells	4 cultures from 4 mice	5 cultures from 5 mice	0.73
2E	% Annexin V/PI-positive cells	4 cultures from 4 mice	5 cultures from 5 mice	0.016
2F	Gfap/DAPI-positive cells Tubb3a/DAPI-positive cells	4 cultures from 4 mice	5 cultures from 5 mice	0.016 (astrocytes); 0.016 (neurons)
2I	Neuron morphology	29 neurons from 4 cultures (7-8 neurons/culture)	30 neurons from 5 cultures (6 neurons/culture)	-
2K	Astrocyte morphology	159 astrocytes from 4 cultures (25-60 astrocytes/culture)	269 astrocytes from 5 cultures (45-60 astrocytes/culture)	0.016
4A	Ki67-positive cells	5 mice	8 mice	0.62
4C	BrdU/NeuN-positive cells	4 mice	4 mice	0.69
5A	% Gfp/Dcx/Calb1-positive cells	3 mice	3 mice	0.40 (immature); 0.10 (mature)
5C	% Calb2/Dcx-positive cells	3 mice	3 mice	0.057
5D	BrdU-positive cells	10 mice	10 mice	0.81
5E	% BrdU/Dcx/Calb-positive cells	8 mice	6 mice	0.087 (immature); 0.0080 (mature)
5G	Neuron morphology	20 neurons from 3 mice	23 neurons from 4 mice	-
5I	Spine numbers	32 neurons from 3 mice (10-12 neurons per mouse)	46 neurons from 4 mice (11-13 neurons per mouse)	< 0.0001
5J	Spine sizes	150 spines from 3 mice (50 spines per mouse)	200 spines from 4 mice (50 spines per mouse)	0.057
5K	% Mushroom spines	150 spines from 3 mice (50 spines per mouse)	200 spines from 4 mice (50 spines per mouse)	0.057
6A	BrdU-positive cells	4 STD; 6 ENR	3 STD; 3 ENR	-
6B	BrdU-positive cells	7 STD; 7 ENR	13 STD; 12 ENR	-
6E	c-Fos in DG after ENR	5 mice	5 mice	0.0079
6F	c-Fos (infrapyramidal)	5 mice	5 mice	0.84
6F	c-Fos (suprapyramidal)	5 mice	5 mice	0.0079
6	% Brdu/NeuN/c-Fos-positive cells	5 mice	5 mice	0.83
6	% c-Fos/Dcx-positive cells	5 mice	4 mice	0.56
6	c-Fos in CA3 after ENR	4 mice	5 mice	0.032
6	% c-Fos/Pv-positive cells	4 mice	4 mice	0.057
7C	Path length	13 mice	23 mice	0.014 (day 4) < 0.0001 (day 5)
7D	Latency	13 mice	23 mice	0.0074 (day 4) 0.0012 (day 5)
7E	% Time in old goal position	13 mice	23 mice	< 0.0001
EV3B	% Gfp/Sox2/S100b-positive cells	3	4	0.63 (NSPCs); 0.23 (astrocytes)
EV3E	Sox2-positive cells	13 mice	15 mice	0.59
EV3G	Ki67-positive cells	7 mice	7 mice	0.80
