# Supplementary material for: De novo DNA methylation controls neuronal maturation during adult hippocampal neurogenesis
Source: EMBO J. 2021 Aug 2;40(18):e107100. doi: 10.15252/embj.2020107100 (PMC8441477; doi:10.15252/embj.2020107100)
Supplement: Supplementary file 3 — Expanded View Figures PDF [file EMBJ-40-e107100-s003.pdf]

Expanded View Figures

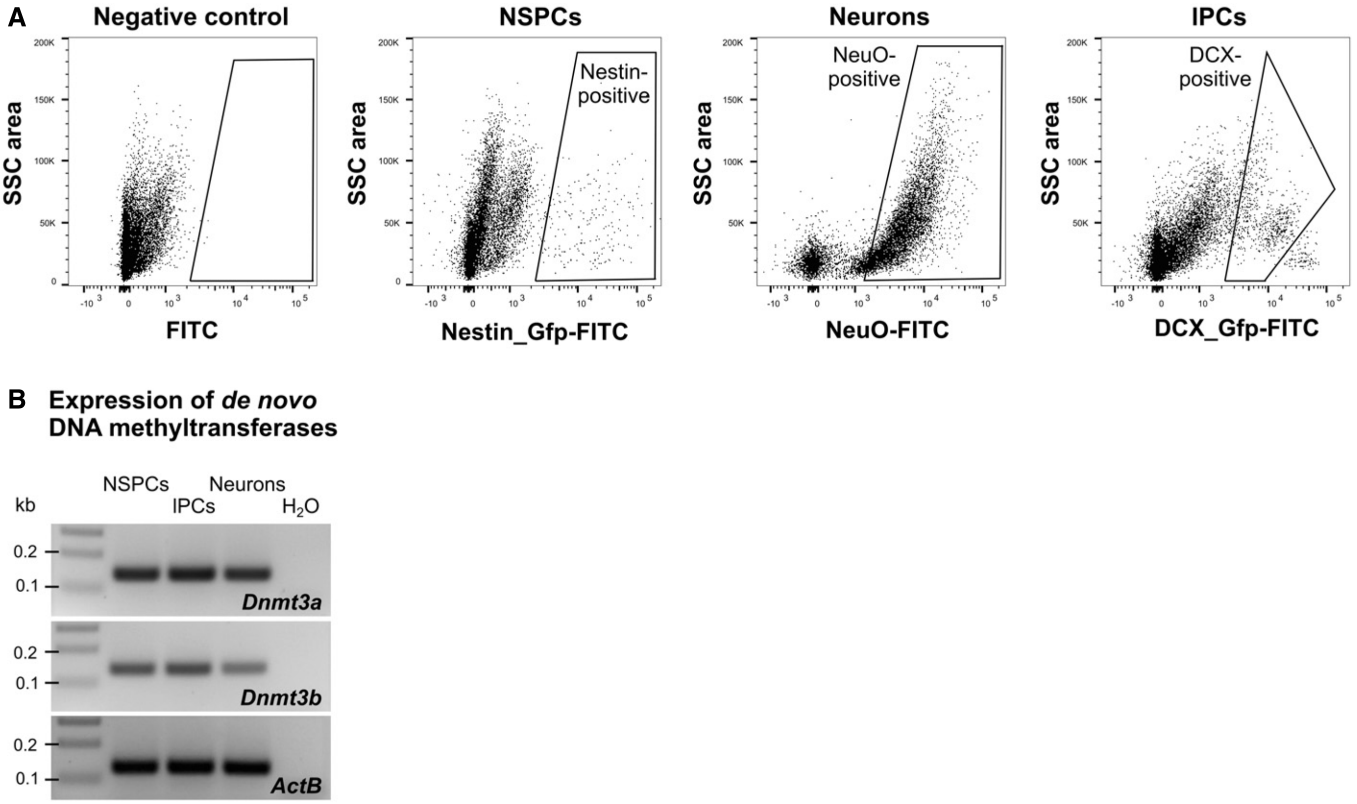

Figure EV1. ***De novo*** **methyltransferases are expressed in different cell stages of adult hippocampal neurogenesis.**

A FACS strategy for isolation of neural stem and early progenitor cells (NSPCs), late progenitor cells (lPCs), and neurons from the adult mouse hippocampus. After separating single cells using their forward scatter (FSC) and side scatter (SSC) properties, dead cells were excluded based on incorporation of propidium iodide. NSPCs were isolated from *Nestin*::EGFP mice and lPCs from *Dcx*::GFP mice based on Gfp intensity. Neurons were identified based on incorporation of the neuron-specific dye NeuroFluor (NeuO).

B Transcript expression of *de novo* DNA methyltransferases *Dnmt3a* and *Dnmt3b* in NSPCs, lPCs, and neurons as determined by RT–PCR.

© 2021 The Authors

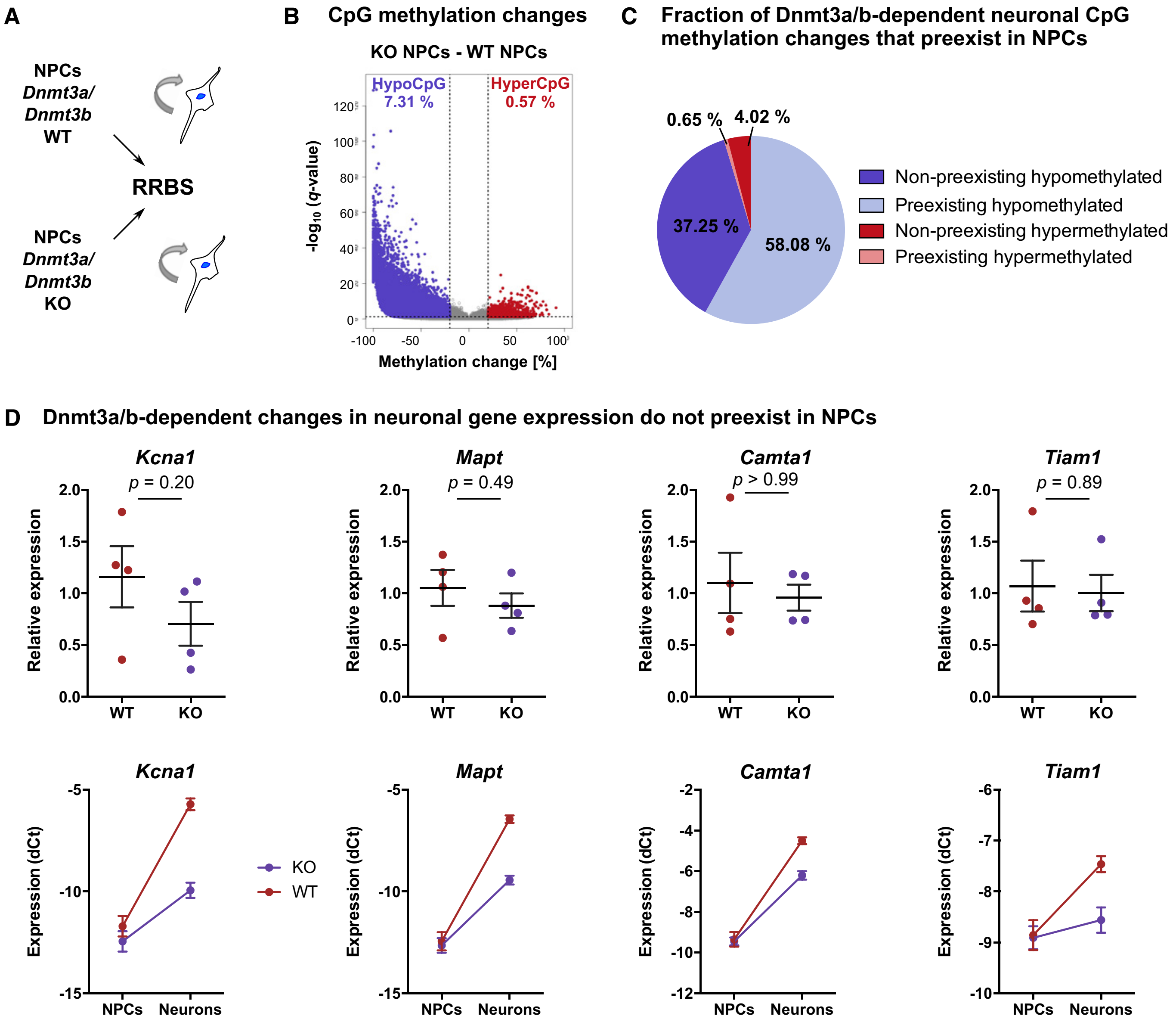

Figure EV2. Dnmt3a/b-dependent CpG methylation and transcriptional changes during differentiation of adult hippocampal NPCs into neurons.

A RRBS was performed on *in vitro* cultures of proliferating NPCs that were derived from the hippocampus of adult *Dnmt3a/b*-WT and KO mice ($n = 3$ cell lines per genotype).

B Significantly differentially methylated CpGs between KO and WT NPCs ($q < 0.05$; absolute methylation differences greater than 20%) are highlighted in violet (hypomethylated CpGs—hypoCpG) and red (hypermethylated CpGs—hyperCpG). Percentages of differentially methylated CpGs among all CpGs covered by RRBS are indicated with the respective color.

C Dnmt3a/b-dependent methylation changes in neurons (see Fig 3B) separated into CpGs that were differentially methylated already in NPCs (preexistent) compared to CpG methylation differences that emerged during the course of neuronal differentiation (non-preexistent). CpGs were further divided into hypomethylated or hypermethylated sites in KO versus WT neurons.

D Dnmt3a/b mediate the transcriptional up-regulation of neuronal genes during neuronal differentiation. Depicted are expression fold changes in KO NPCs versus WT NPCs (top) and normalized expressions (normalized to *Actb*) in NPCs and neurons (bottom). Depicted *P*-values are from Mann–Whitney test ($n = 4$ cell lines per genotype). Depicted are data points for every culture with genotype means ± SEM (top) or genotype means ± SEM (bottom).

© 2021 The Authors

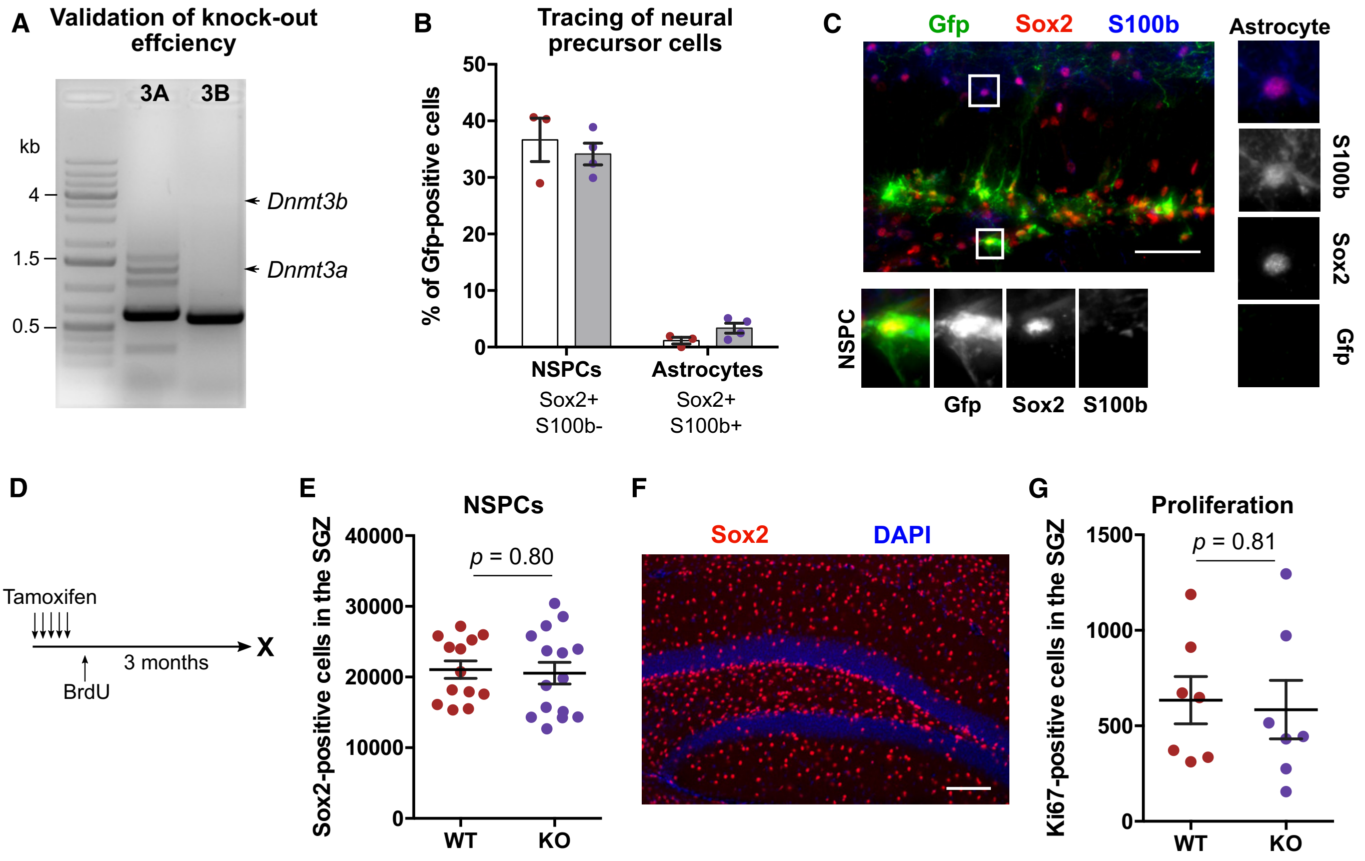

Figure EV3. Deletion of *de novo* DNA methyltransferases does not influence long-term maintenance of adult hippocampal NSPCs *in vivo*.

- A Validation of genomic deletion of *Dnmt3a* (3A) and *Dnmt3b* (3B) in Gfp-positive cells by polymerase chain reaction. Arrows indicate expected sizes for wildtype *Dnmt3a* and *Dnmt3b* amplicons.
- B No difference in the percentage of NSPCs and astrocytes among Gfp-positive cells was detected between WT (red; n = 3 mice) and KO mice (violet; n = 4 mice). Corresponding experimental scheme is depicted in Fig 4E. Depicted are data points for every animal with genotype means ± SEM.
- C Representative fluorescent image for detection of NSPCs and astrocytes. Depicted astrocyte is Gfp-negative. Scale bar: 50 µm.
- D Experimental outline for results presented in E-G and in Fig 5D and E.
- E No difference was found in the total numbers of NSPCs in the subgranular zone (SGZ) between WT and KO mice 3 months after tamoxifen administration (n = 13 mice, WT; n = 15 mice, KO). Depicted are data points for every animal with genotype means ± SEM (P-value from unpaired t-test).
- F Representative fluorescent image for analysis of Sox2-positive NSPCs. Scale bar: 100 µm.
- G No difference in the total numbers of proliferating cells in the SGZ between WT and KO mice 3 months after recombination (n = 7 mice per genotype). Depicted are data points for every animal with genotype means ± SEM (P-value from unpaired t-test).

© 2021 The Authors

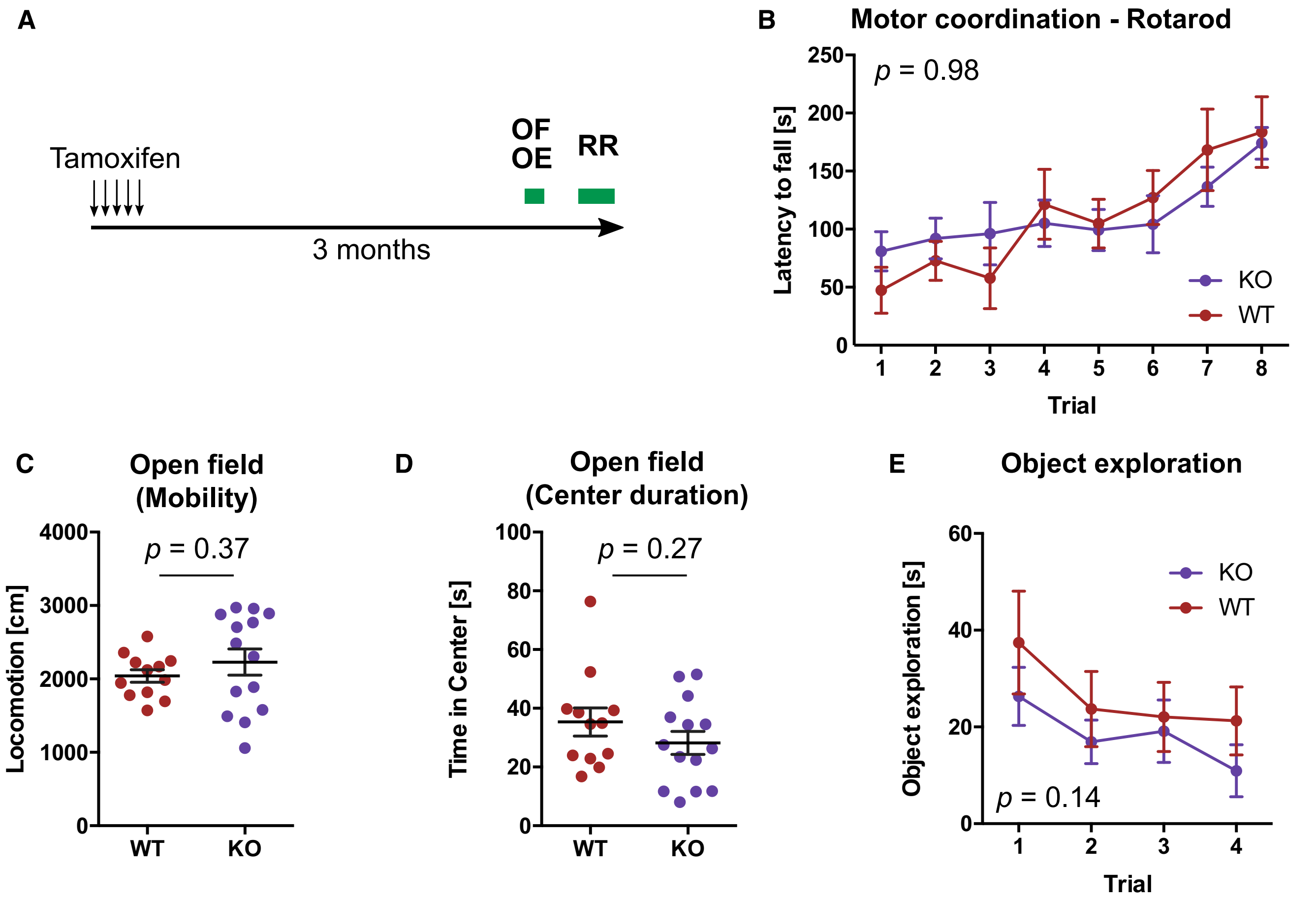

Figure EV4. Deletion of *Dnmt3a* and *Dnmt3b* in adult NSPCs and their progeny does not influence motor coordination or exploratory activity.

A Three months after administration of tamoxifen, mice were tested in one trial of open field test (OF) followed by four trails of object exploration test (OE). One week later, mice were tested on the rotarod (RR).

B Rotarod performance in WT and KO mice. Depicted *P*-value corresponds to genotype effect from non-parametric longitudinal model (n = 7, WT; n = 11, KO). Depicted are means ± SEM.

C Distance mice moved in the open field (n = 12, WT; n = 14, KO; *P*-value from Mann–Whitney test). Depicted are data points per animal with genotype means ± SEM.

D Time mice spent in the center of the open field (n = 12, WT; n = 14, KO; *P*-value from Mann–Whitney test). Depicted are data points per animal with genotype means ± SEM.

E Time mice spent around objects. In trial 4, one object was replaced with a novel object. Depicted *P*-value corresponds to genotype effect from non-parametric longitudinal model (n = 7, WT; n = 11, KO). Depicted are means ± SEM.

 © 2021 The Authors
