# Supplementary material for: De novo DNA methylation controls neuronal maturation during adult hippocampal neurogenesis
Source: EMBO J. 2021 Aug 2;40(18):e107100. doi: 10.15252/embj.2020107100 (PMC8441477; doi:10.15252/embj.2020107100)
Supplement: Supplementary file 1 — Review Process File [file EMBJ-40-e107100-s002.pdf]

Foxm1 regulates neural progenitor fate during spinal cord regeneration

Diane Pelzer, Lauren Phipps, Raphael Thuret, Carlos Gallardo-Dodd, Syed Murtuza Baker, and Karel Dorey
DOI: 10.15252/embr.202050932

Corresponding author(s): Karel Dorey (karel.dorey@manchester.ac.uk)

Review Timeline:

Transfer from Review Commons:	20th May 20
Editorial Decision:	26th May 20
Revision Received:	13th May 21
Editorial Decision:	23rd Jun 21
Revision Received:	24th Jun 21
Accepted:	1st Jul 21

Editor: Esther Schnapp

Transaction Report: This manuscript was transferred to EMBO Reports following peer review at Review Commons.

(Note: With the exception of the correction of typographical or spelling errors that could be a source of ambiguity, letters and reports are not edited. Depending on transfer agreements, referee reports obtained elsewhere may or may not be included in this compilation. Referee reports are anonymous unless the Referee chooses to sign their reports.)

1st Editorial Decision 26th May 2020

Dear Dr. Dorey,

Thank you for the submission of your manuscript with referee reports from Review Commons and your proposed point by point response to EMBO reports.

Given the positive reports, and your clear proposal for how to address all points, I am happy to invite you to address all points as you suggest and submit a revised manuscript to EMBO reports.

Please also address all referee concerns in a complete point-by-point response. Acceptance of the manuscript will depend on a positive outcome of a second round of review. It is EMBO reports policy to allow a single round of major revision only and acceptance or rejection of the manuscript will therefore depend on the completeness of your responses included in the next, final version of the manuscript.

Revised manuscripts should be submitted within three months of a request for revision; they will otherwise be treated as new submissions. Please contact us if a 3-months time frame is not sufficient for the revisions so that we can discuss this further. You can either publish the study as a short report or as a full article. For short reports, the revised manuscript should not exceed 27,000 characters (including spaces but excluding materials & methods and references) and 5 main plus 5 expanded view figures. The results and discussion sections must further be combined, which will help to shorten the manuscript text by eliminating some redundancy that is inevitable when discussing the same experiments twice. For a normal article there are no length limitations, but it should have more than 5 main figures and the results and discussion sections must be separate. In both cases, the entire materials and methods must be included in the main manuscript file.

Regarding data quantification, please specify the number "n" for how many independent experiments were performed, the bars and error bars (e.g. SEM, SD) and the test used to calculate p-values in the respective figure legends. This information must be provided in the figure legends. Please also include scale bars in all microscopy images.

IMPORTANT NOTE: we perform an initial quality control of all revised manuscripts before re-review. Your manuscript will FAIL this control and the handling will be DELAYED if the following APPLIES:
1) A data availability section providing access to data deposited in public databases is missing. If you have not deposited any data, please add a sentence to the data availability section that explains that.
2) Your manuscript contains statistics and error bars based on n=2 or on technical replicates. Please use scatter blots in these cases. No statistics can be calculated if n=2.

When submitting your revised manuscript, please carefully review the instructions that follow below. Failure to include requested items will delay the evaluation of your revision.

1) a .docx formatted version of the manuscript text (including legends for main figures, EV figures and tables). Please make sure that the changes are highlighted to be clearly visible.

2) individual production quality figure files as .eps, .tif, .jpg (one file per figure).
See https://wol-prod-cdn.literatumonline.com/pb-assets/embo-site/EMBOPress_Figure_Guidelines_061115-1561436025777.pdf for more info on how to prepare your figures.

3) We replaced Supplementary Information with Expanded View (EV) Figures and Tables that are collapsible/expandable online. A maximum of 5 EV Figures can be typeset. EV Figures should be cited as 'Figure EV1, Figure EV2" etc... in the text and their respective legends should be included in the main text after the legends of regular figures.

- For the figures that you do NOT wish to display as Expanded View figures, they should be bundled together with their legends in a single PDF file called *Appendix*, which should start with a short Table of Content. Appendix figures should be referred to in the main text as: "Appendix Figure S1, Appendix Figure S2" etc. See detailed instructions regarding expanded view here: <https://www.embopress.org/page/journal/14693178/authorguide#expandedview>

- Additional Tables/Datasets should be labeled and referred to as Table EV1, Dataset EV1, etc. Legends have to be provided in a separate tab in case of .xls files. Alternatively, the legend can be supplied as a separate text file (README) and zipped together with the Table/Dataset file.

4) a .docx formatted letter INCLUDING the reviewers' reports and your detailed point-by-point responses to their comments. As part of the EMBO Press transparent editorial process, the point-by-point response is part of the Review Process File (RPF), which will be published alongside your paper.

5) a complete author checklist, which you can download from our author guidelines <https://www.embopress.org/page/journal/14693178/authorguide>. Please insert information in the checklist that is also reflected in the manuscript. The completed author checklist will also be part of the RPF.

6) Please note that all corresponding authors are required to supply an ORCID ID for their name upon submission of a revised manuscript (<https://orcid.org/>). Please find instructions on how to link your ORCID ID to your account in our manuscript tracking system in our Author guidelines <https://www.embopress.org/page/journal/14693178/authorguide#authorshipguidelines>

7) Before submitting your revision, primary datasets produced in this study need to be deposited in an appropriate public database (see https://www.embopress.org/page/journal/14693178/authorguide#datadeposition). Please remember to provide a reviewer password if the datasets are not yet public. The accession numbers and database should be listed in a formal "Data Availability" section placed after Materials & Method (see also https://www.embopress.org/page/journal/14693178/authorguide#datadeposition). Please note that the Data Availability Section is restricted to new primary data that are part of this study. * Note - All links should resolve to a page where the data can be accessed. *
If your study has not produced novel datasets, please mention this fact in the Data Availability Section.

8) We would also encourage you to include the source data for figure panels that show essential data. Numerical data should be provided as individual .xls or .csv files (including a tab describing the data). For blots or microscopy, uncropped images should be submitted (using a zip archive if multiple images need to be supplied for one panel). Additional information on source data and instruction on how to label the files are available at <https://www.embopress.org/page/journal/14693178/authorguide#sourcedata>.

9) Our journal also encourages inclusion of *data citations in the reference list* to directly cite

datasets that were re-used and obtained from public databases. Data citations in the article text are distinct from normal bibliographical citations and should directly link to the database records from which the data can be accessed. In the main text, data citations are formatted as follows: "Data ref: Smith et al, 2001" or "Data ref: NCBI Sequence Read Archive PRJNA342805, 2017". In the Reference list, data citations must be labeled with "[DATASET]". A data reference must provide the database name, accession number/identifiers and a resolvable link to the landing page from which the data can be accessed at the end of the reference. Further instructions are available at https://www.embopress.org/page/journal/14693178/authorguide#referencesformat

We would also welcome the submission of cover suggestions, or motifs to be used by our Graphics Illustrator in designing a cover.

As part of the EMBO publication's Transparent Editorial Process, EMBO reports publishes online a Review Process File (RPF) to accompany accepted manuscripts. This File will be published in conjunction with your paper and will include the referee reports, your point-by-point response and all pertinent correspondence relating to the manuscript.

You are able to opt out of this by letting the editorial office know (emboreports@embo.org). If you do opt out, the Review Process File link will point to the following statement: "No Review Process File is available with this article, as the authors have chosen not to make the review process public in this case."

I look forward to seeing a revised version of your manuscript when it is ready. Please let me know if you have questions or comments regarding the revision.

Kind regards,
Esther

Esther Schnapp, PhD
Senior Editor
EMBO reports

Review #1

1. How much time do you estimate the authors will need to complete the suggested revisions:

Estimated time to Complete Revisions (Required)

(Decision Recommendation)

Between 1 and 3 months

2. Evidence, reproducibility and clarity:

Evidence, reproducibility and clarity (Required)

This manuscript describes the role of foxm1 in the regeneration of the Xenopus tadpole tail. Foxm1 is identified as upregulated in a well described and analysed single cell RNA seq of regenerating tails. In crispants (CRISPR/cas9 injected tadpoles in which much of foxm1 is mutated), the tail is regenerated, but there is a lower number of neurons. The data is overall well analysed and presented. **Major comments:** Although the authors performed some experiments to reveal the signalling pathway upstream of foxm1, including the inhibition of the sonic hedgehog pathway. Looking at the proliferation patterns in figure 4, it looks like the ventral, floor plate like domain is disturbed in foxm1 mutants. Is it possible that foxm1 is UPstream of shh signalling, rather than downstream? Showing the expression of hh pathway genes (e.g. patched, olig2) in the wt and mutant spinal cords might further inform about the position of foxm1 in the signalling cascade controlling regenerative neurogenesis. Higher magnifications of the tissue sections in figure 4 need to be shown. The in situ signal and distribution of cells is not well visible. Do the experiments on page 7 show that there is no difference in cell cycle length, or may there (also) be changes in the number of cells entering the cycle? A number of results are only borderline significant (e.g. p=0.04). It is not clear how group sizes were determined and what the power of the experiments is. **Minor comments:** dendogram should be dendrogram throughout Abstract: "reduction in THE NUMBER OF neurons" top of page 7: sequencing "revealed" (not leading to)

3. Significance:

Significance (Required)

The main finding of this manuscript is that foxm1 has a regeneration-specific role and rather than control progenitor proliferation, it is important for neurogenesis/neuronal maturation. This is an interesting finding, of wider relevance for researchers in regenerative neurogenesis and stem cell differentiation. It appears that with relatively few more experiments, the position of foxm1 in the pro-neurogenesis cascade could be more clearly determined, in

particular, the effect of its mutation on the hh pathway. Own expertise: regeneration, neurogenesis REFEREES CROSS COMMENTING Reading over our reviews, it looks like we all have the same concerns (in so many words) 1. that the cell cycle conclusions are not fully supported by the data and 2. that the disorganisation of the spinal ependymal zone/proliferating cells is insufficiently characterised. Both can be addressed experimentally and we have made suggestions how. What else would be important to strengthen the manuscript?

Review #2

1. How much time do you estimate the authors will need to complete the suggested revisions:

Estimated time to Complete Revisions (Required)

(Decision Recommendation)

Between 3 and 6 months

2. Evidence, reproducibility and clarity:

Evidence, reproducibility and clarity (Required)

Summary: In the manuscript entitled "Foxm1 regulates neuronal progenitor fate during spinal cord regeneration "Pelzer and colleague described molecular events occurring during spinal cord regeneration in Xenopus tadpole. Using a transcriptomic approach, they show that the transcription factor Foxm1 is specifically and temporally upregulated in the spinal cord during regeneration. Foxm1 knockdown using CRISPR/cas9 technology reduces spinal regeneration indicating a requirement of this transcription factor in this process. Using single cell transcriptomic analyses, they show that Foxm1 is expressed only during early regeneration in a specific cluster of neural progenitor cells; these cells display upregulation of several genes related to the cell cycle and half of them are in S phase suggesting that they have specific proliferative features. Eventually, they show that Foxm1 controls the proliferation/differentiation balance of neural progenitor cells. **Major comments:** *Are the key conclusions convincing?* Yes, the key conclusions summarized above are convincing. The conclusions that deserve to be reinforced are those relating to the cell cycle length (see below). *Should the authors qualify some of their claims as preliminary or speculative, or remove them altogether?* The statement based on the pulse-chase experiments (Figure 2F) that Foxm1 does not affect the overall length of the cell cycle is too strong. The same result could be obtained with a change in cell cycle length combined with self-renewing rather than differentiating divisions. A measure of the total cell cycle length would remove this ambiguity. Knowing that the total cell cycle length is not modified would also allow to conclude that at 3dpa the increase of the cells in S phase reflects a longer S phase. The same result would be obtained going from 45,7% at 3dpa to 12% at 5 dpa without changing the S phase length if the total cell cycle length increases 4 times between 3dpa and 5dpa. Literature data reporting cell cycle length at 3dpa and 5dpa would be sufficient to

verify the credibility of the latter hypothesis. The authors claim that Foxm1 affects neurogenesis independently of its ability to control the proliferation rate contrary to what has been observed during Xenopus primary neurogenesis (Ueno et al., 2008). In their paper, Ueno and colleagues (2008) knock down Foxm1 in primary neurogenesis using RNAi and show a strong reduction of the number of mitotic cells using PH3 labelling and the total number of cells stained with Hoechst. Foxm1 controlling the G2/M transition it is an obvious readout. It would be useful to determine whether downregulating Foxm1 affects mitosis in regenerative spinal cord using PH3 labelling for example. If it is not the case, it should be verified that CRISPR/Cas9 that leads to 52% reduction of Foxm1 expression mimics the effect of RNAi at early stage inducing a reduction of PH3 cells in primary neurogenesis. *Would additional experiments be essential to support the claims of the paper?* Measurement of the total cell cycle length using cumulative EdU for example or BrdU/EdU dual labelling in Sox3+ cells will allow to show whether or not Foxm1 impacts on cell cycle length. Quantifying PH3 cells in primary neurogenesis as performed by Ueno and colleagues and in regeneration following CRISPR/Cas9 knockdown will allow to conclude whether the mechanisms are different. Note that PH3 detect cells in early mitotic phases that is more difficult with DAPI staining. *Are the suggested experiments realistic in terms of time and resources? It would help if you could add an estimated cost and time investment for substantial experiments.* We are not familiar enough with experiments in Xenopus to give a clear answer. Measurement of cell cycle features can be time consuming. What is important here and is not mentioned is the total cell cycle length of regenerative neural progenitor cells. If it has been measured in other studies it should be mentioned at 3dpa and 5dpa because it will also help for interpretation of the data. Cumulative EdU experiments in a context of short cell cycle length (around 10 hours) is feasible. *Are the data and the methods presented in such a way that they can be reproduced?* In general yes. I would appreciate to have a precision regarding EdU treatment. It is written: "For EdU labelling, NF50 tadpoles at 3dpa were injected with 3 times 4.2nL of 10mM EdU (Life Technologies) in DMSO for 3hr". Is it 3 times an hour for a total of 3 hours? *Are the experiments adequately replicated and statistical analysis adequate?* yes **Minor comments:** -Specific experimental issues that are easily addressable. Can the authors comment on the detection of S phase markers in neurons? Figure 3F (right side bar; neur.) The main defect observed following Foxm1 downregulation is the disorganization of the structure (figure 4B) including in the anterior spinal cord area. The authors should comment on that. Is there ectopic mitosis in that context? *-Are prior studies referenced appropriately?* yes *-Are the text and figures clear and accurate?* Abstract: I do not understand the end: "In foxm1-/- tadpoles, we observed a reduction of neurons in the regenerating spinal cord, suggesting that neuronal differentiation is necessary for the regenerative process. Altogether, our data show that different tissues respond differently to injury and reveal a new role for neuronal differentiation during regeneration." What tissues are they referring to in the last sentence that were analyzed here? Paragraph Foxm1 regulates the fate ... "We next analysed Sox3 expression by immunofluorescence using anti-Sox3 ... As expected, in the non-regenerating spinal cord Sox3 is expressed in the cells lining the ventricle. We also observed Sox3+ extensions into the mantle zone of the spinal cord (Figure 4B, white arrowheads)." Why do the authors state that it is in the mantle zone? It would be useful to have a marker for differentiating neurons to visualize the mantle zone. "In contrast, the proportion of self-renewal divisions increases significantly in Foxm1-/- tadpoles". What are the different mode of divisions in regenerative spinal cord? Self-renewal, asymmetric neurogenic and terminal neurogenic like in a developing spinal cord? Can the authors exclude also asymmetric neurogenic divisions if they exist? Some pictures should be improved: PCNA labelling Fig S3-E should be better-quality. The authors claim that they identified S versus G1/G2 phases using PCNA labelling

that of course is possible but difficult to visualize on the pictures. A better resolution of the pictures would be more convincing. *-Do you have suggestions that would help the authors improve the presentation of their data and conclusions?* If the data in the literature permit, more information on what is known about the characteristics of the cell cycle during spinal cord regeneration in amphibians can be provided.

3. Significance:

Significance (Required)

-Describe the nature and significance of the advance (e.g. conceptual, technical, clinical) for the field. RNAseq data is clearly a resource for the scientific community. Moreover, this work extends our knowledge of Foxm1 function in primary neurogenesis to tadpole spinal cord regeneration. *-Place the work in the context of the existing literature (provide references, where appropriate)* The research of the molecular cues responsible for controlling the choice between proliferation and differentiation, the role of the cell cycle in this process in the developing or regenerating nervous system is clearly of interest. - State what audience might be interested in and influenced by the reported findings. Neurobiologists, cell biologists particularly those working in the role of the cell cycle in cell fate, and people interest in the process of regeneration including those working on stem cells. *-Define your field of expertise with a few keywords to help the authors contextualize your point of view. Indicate if there are any parts of the paper that you do not have sufficient expertise to evaluate.* Neurodevelopment, cell cycle, neurogenesis, spinal cord, vertebrate Lower expertise: regeneration, Xenopus, RNAseq single cell analyses

Review #3

1. How much time do you estimate the authors will need to complete the suggested revisions:

Estimated time to Complete Revisions (Required)

(Decision Recommendation)

Less than 1 month

2. Evidence, reproducibility and clarity:

Evidence, reproducibility and clarity (Required)

The main aim of this study is to investigate the role of Foxm1 during spinal cord regeneration in Xenopus tropicalis. The authors first identified Foxm1 as the top upregulated gene in the regenerating spinal cord tissue by bulk RNA-seq analysis. Interestingly, they found that knocking out Foxm1 resulted in regeneration defect of the tail, while the proportion of spinal cord progenitor cells in S phase in the regenerating spinal cord was not affected. The authors

then compared the proportion of cells in different cell cycle stages between uninjured and regenerating spinal cord and found that there was a significant increase in the proportion of S phase in the 3-day post-amputated spinal cord, which correlated with the expression timing of Foxm1. Lastly, they showed that the progenitor pool is increased, and the neuron pool decreased, in the regenerating spinal cord in the absence of Foxm1. This observation lead to the hypothesis that Foxm1 might play roles in controlling the fate switch from progenitors to neuron, a key feature of regenerative species. Overall, it is laudable that the authors took on this challenging topic with state-of-the-art scRNA-seq analysis and took time to generate Foxm1 knock-out frog line with CRISPR/Cas9 technology. However, there are several points the authors should address in the study to make a convincing story. The authors also have to be more cautious about interpretation of the data (see below). It is intriguing that frogs can survive without Foxm1, as it has been reported that Foxm1 knock-out mice is embryonic lethal. Therefore, the authors should confirm that foxm1 transcript and protein are indeed absent in foxm1-null tadpoles with ISH and IHC on the cross-section of tail. It was not shown in the manuscript. This is important because the deletion is made in the beginning of the coding sequence but there is an ATG present just after the deletion, so initiation from this downstream methionine may be occurring. In addition, whether the same regeneration phenotype exists in foxm1-null tadpoles as in F0 is also not mentioned or shown. The documentation of the regeneration phenotype should be in more detail in addition to length. This is important because, if the foxm1-null tadpoles also have the regeneration phenotype, comparing the progenitor cells with cross section in Figure4B would be tricky. For example, the morphology of cells near the amputation plane and those near the terminal vesicle is very different even in wildtype. Without knowing the corresponding regions of the regenerating spinal cords between wildtype and foxm1-null tadpoles, it is difficult to make a meaningful comparison. The authors state at the end of second section "...Foxm1 does not affect the overall length of cell cycle" based on the one-time EdU pulse labeling (Figure2D-F). The data only shows the percentage of cells in S-phase is the same but does not tell us about the length of cell cycle. In order to make this claim, the author would have to do multiple EdU pulse labeling and deduce the cell cycle length from the saturation curve, as describe in Nowakowski et al. 1989. This is important as the main conclusion of this manuscript is based on this observation. The fact that the ratio of Edu positive spinal cord progenitors remains the same (Figure2E) and that the shortening of regenerating spinal cord (Figure 2B) does not come together and require more explanation. It goes back to the previous point of incomplete documentation of the regeneration phenotype. In the third section about scRNA-seq analysis, the authors state that "...whilst some cell types cluster together, neural progenitors display a big shift in their transcriptome" based on the tSNE plot in Figure3D. However, it is also likely due to batch effect, which is common in scRNA-seq data. The authors could computationally correct the batch effect with the Seurat or Harmony package. However, to completely exclude the possibility of batch effect, the authors would have to do multiplexing of these two samples using MULTI-seq or CITE-seq technique before making a definitive conclusion. In general, caution has to be taken when naming a cluster by a single marker. It would be a better measure to show the unbiased clustering result of Figure 3D and to see if foxm1 is the representative marker of this cluster. It is also not advisable to define a cluster with cell cycle-related genes, as it represents a cell state rather than a cell type. On the same note, it is not surprising that foxm1+ cells are mostly in S and G2/M phase as shown in Figure3F because foxm1 is known to be highly expressed in S and G2/M phase. In figure4C and figure 4E, the authors showed progenitor pool increased and neuron pool decreased. There are other possible explanations in addition to the failure of neuron differentiation as the authors suggested. It can be that the progenitors are over proliferating, or the neurons are dying. Different possibilities should be tested in order to make a strong conclusion. It will be

helpful for the readers by showing the representative image of mty1 staining and to show the absolute number of counted cells in addition to percentages. To sum up, the manuscript requires a major improvement to strengthen the finding and novelty. One possible direction is to delineate the molecular pathways of progenitor-to-neuron transition with their scRNA-seq data in combination with in vivo validation. For example, trajectory analysis could give us insights about how the transition happens, and which cells express foxm1 and when. The authors listed the potential downstream factors in figureS2C, but unfortunately did not pursue this direction. The authors should also take advantage of their foxm1-null frog line to compare the scRNA-seq data between wildtype and foxm1-null tadpoles during tail regeneration. There are some minor comments following the order of Figures: •In order to make a strong statement on the "spinal cord-only" expression of Foxm1, Foxm1 ISH or IHC on the cross-section of the regenerate is appreciated (Figure1E). •The statistics is missing in Figure1F. •Figure3F and Figure 4D require more explanation or the figures have to be simplified. •It was not mentioned how CRISPR/Cas9 knock-down (Figure4A) was performed. Whether the term "knock-down" means mosaic F0 or heterozygous F1, is unclear. •The quality of IF images should be improved (Figure4B). •Figure legends of the supplementary Figure1E-I are missed labeled to FigureS2I-E.

3. Significance:

Significance (Required)

This is one of a very small number of manuscripts that successfully male CRISPR-induced mutants to study regeneration in Xenopos. The authors even report F1 homozygous animals. This would be of great interest to the community. Unfortunately the work verifying the null mutation is lacking and more experiments with the F l homozygotes are needed. A better molecular definition of the phenotype would be needed to attain the highest potential of excitement for this work. Expertise: regeneration, genome engineering REFEREES CROSS COMMENTING A strange part of the manuscript is that the growth defect is only shown using f0 transgenics. In my view, they need to clarify and quantitate the major phenotypes including the spinal cord growth defects using the f1 homozygotes that exist.

Detailed response to the reviewers

Reviewer #1

1- S/he was wondering whether Foxm1 might be upstream of Shh signalling rather than downstream.
Shh is known to be important for tail regeneration much earlier than the onset of *foxm1* expression (Taniguchi et al. 2014; Hamilton et al. 2021). Furthermore, impairing Shh signalling during tail regeneration affects proliferation in the spinal cord, a phenotype not consistent with the one we observed for Foxm1 (Taniguchi et al. 2014). We agree that the role of Shh in neuronal differentiation during regeneration is an interesting question but we felt it was outside the scope of this manuscript.

2- Higher magnifications of the tissue sections in figure 4 need to be shown.
We apologise for the fact that the pdf compression led to a loss of resolution. This has been rectified throughout the manuscript.

3- the reviewer asked if there is no difference in cell cycle length, or may there (also) be changes in the number of cells entering the cycle?
We have now shown by Dual Pulse Labelling that there is no change in the duration of the overall cell cycle length between wild type and mutant tadpoles (Fig. 3G/H). We have also shown that the percentage of cycling Sox3+ve cells (PCNA+) at 3dpa is the same independently of the genotype (Appendix FigS1). It is therefore very unlikely that our results could be explained by a change in the number of cells entering the cycle.

4- A number of results are only borderline significant (e.g. p=0.04). It is not clear how group sizes were determined and what the power of the experiments is.
The main result with a borderline significance is the regrowth of the tail when comparing wild type and knockdown tadpoles. This might be due to a subtle phenotype or the fact that these experiments were done on knockdown tadpoles rather than knockout. The new data on tail regeneration on knockout now has a p_value<0.0001. The other experiment with a small significance, is the RT-qPCR in Figure 4A (new Figure EV5A), also done comparing the knockdown and wild type. However, in this case we feel that the protein work (immunofluorescence on Figure 5A-F) supports the qPCR data and is much more revealing in characterising the phenotype of the *foxm1* mutant.
In general, all experiments were done from three independent crosses with at least three different animals per cross.

5- minor comments

- *dendogram should be dendrogram throughout:* this has been change
- *Abstract: "reduction in THE NUMBER OF neurons"* : corrected
- *top of page 7: sequencing "revealed" (not leading to)*: corrected

Reviewer #2

1- *Measurement of the total cell cycle length using cumulative EdU for example or BrdU/EdU dual labelling in Sox3+ cells will allow to show whether or not Foxm1 impacts on cell cycle length.*
We have done these experiments (see above) and they do not show differences in overall length between mutant and wild type tadpoles.

2- *Quantifying PH3 cells in primary neurogenesis as performed by Ueno and colleagues and in regeneration following CRISPR/Cas9 knockdown will allow to conclude whether the mechanisms are different.*

We have performed these experiments by comparing the proliferation in the neural plate at NF13 in embryos injected with either a morpholino preventing the splicing of *foxm1* RNA or our Cas9/gRNA against the *foxm1* locus used to generate the knockout. In all conditions, we do not observe a difference in proliferation quantified using anti-pH3 staining. These data suggest that in *Xenopus tropicalis,* Foxm1 does not regulate the proliferation of neuronal progenitor cells.

3- The reviewer wanted precision regarding EdU treatment. This is now explicitly described in the methods section: we do one injection of 12.6nL (3 times 4.2nL), then chased for 2 days.

4- minor comments:

- *Can the authors comment on the detection of S phase markers in neurons? Figure 3F (right side bar; neur.)*: we have corrected an error: some bars labelled neurons were in fact differentiated neurons. To clarify this figure (as requested by reviewer 3 also), we have now indicated which bar corresponds to which cluster (Figure 4B). There are still some cells in S/G2/M phases in the neurons cluster, likely due to the presence of some *sox2+* cells in these cluster (Figure 3D)
- *The main defect observed following Foxm1 downregulation is the disorganization of the structure (figure 4B) including in the anterior spinal cord area. The authors should comment on that. Is there ectopic mitosis in that context?:* whilst we agree that this is the case for the regenerating spinal cord, we do not think that the anterior (non-regenerating) spinal cord architecture is different in *foxm1-/-*. We do not see differences in term of the number of Sox3+, Myt1+ and EdU+ cells. Furthermore the distribution of DAPI and Sox3 cells along the dorsoventral axis is similar in wild type and mutants in the non-regenerating spinal cord (Figure EV5C-D). Figures 2D and 5E shows very similar architecture of the non-regenerating spinal cord in mutant and wild type tadpoles. We think that the apparent difference in Figure 4B (new Figure 5A) is due to the deformation of the tissue during the sectioning process.
- *Abstract: I do not understand the end: "In foxm1-/- (…) data show that different tissues respond differently to injury and reveal a new role for neuronal differentiation during regeneration." What tissues are they referring to in the last sentence that were analyzed here?* The last sentence of the abstract refers to the fact we do not observe an upregulation of *foxm1* expression and its transcriptional targets when the whole tail is analysed (Fig.1D), suggesting that we are reporting a spinal cord – specific response. We have changed the sentence to “Altogether, our data uncover a spinal cord – specific response to injury and reveal a new role for neuronal differentiation during regeneration”, removing the reference to other tissues.
- *Paragraph Foxm1 regulates the fate " We also observed Sox3+ extensions into the mantle zone of the spinal cord (Figure 4B, white arrowheads)." Why do the authors state that it is in the mantle zone? It would be useful to have a marker for differentiating neurons to visualize the mantle zone*. This has been done using anti-acetylated Tubulin antibodies (Figure 5B)
- *"In contrast, the proportion of self-renewal divisions increases significantly in Foxm1-/- tadpoles". What are the different mode of divisions in regenerative spinal cord? Self-renewal, asymmetric neurogenic and terminal neurogenic like in a developing spinal cord? Can the authors exclude also asymmetric neurogenic divisions if they exist?* This is an interesting question but at this stage we cannot differentiate between PP, PN and NN divisions. Here, we consider any cells having incorporated EdU and still expressing Sox3 at the end of the chase period as a self-renewal division (i.e. remains a progenitor after division).
- *Some pictures should be improved:* this has been done (see response to reviewer 1).
- *If the data in the literature permit, more information on what is known about the characteristics of the cell cycle during spinal cord regeneration in amphibians can be provided:* changes in cell cycle dynamics have been well described in Axolotl but nothing

has been shown in the Xenopus regenerating spinal cord. We have referenced the Axolotl work and our data are in agreement with theirs.

-

Reviewer #3

1- the reviewer wanted us to confirm that our Crispr/Cas9 model is a true knockout.
We agree with the reviewer that we needed to characterise the *foxm1* KO much better. We have now performed RT-qPCR to quantify precisely the expression of *foxm1* during regeneration in wildtype and mutant tadpoles (Figure EV2E). These data show more than 80%reduction of *foxm1* in the knockout compared to wildtype. We have also generated F2 full knockout tadpoles and show that they display impaired regeneration (Figure 2A/B, see response to reviewer 1). We would love to do IHC but we have not found an antibody against Foxm1 that works in Xenopus yet.

2- The reviewer asked for a better documentation of the regeneration phenotype, in particular asking how we identified equivalent area of regeneration in the wild type versus the mutant tadpoles
We have added more details in the methods, in brief we mark the amputation plane during the embedding process to be able to identify it when sectioning. We then only consider the first 10 sections posterior to amputation plane (120μm), to avoid the neural ampulla which is quite different as the reviewer noted. Furthermore, there is not really a growth phenotype at 3dpa, allowing us to compare proliferation, differentiation etc in the mutant an. d wildtype situation

3- S/he asked us to quantify the length of the cell cycle.
See response to reviewer 1, we have performed these experiments using Dual Pulse Labelling as described in Thuret et al. 2015.

4-The fact that the ratio of Edu positive spinal cord progenitors remains the same (Figure2E) and that the shortening of regenerating spinal cord (Figure 2B) does not come together and require more explanation. It goes back to the previous point of incomplete documentation of the regeneration phenotype. We agree with the reviewer that this present us with an apparent paradox. We suggest in the manuscript that this might have to do with a loss of directionality of division. It has been clearly shown in Axolotl that the main driver for growth of the tail in the fact that upon amputation, division align along the anteroposterior axis. We show here that the distribution of cells along the AP axis is different in wild type and mutants in the regenerate (Fig EV5 C-D), providing a possible explanation for this paradox.

5- The review asked us to correct our scRNAseq for batch effect.
We would like to thank the reviewer for pointing this out and we have tested multiple batch corrections algorithm (Figure EV4A-B). This has led us to identify two clusters specific for 3dpa: a neuronal cluster characterised by the expression of *leptin* (and reported in Aztekin et al. 2019 and Kakebin et al. 2020) and a progenitor cluster expressing *foxm1.*

6- *The reviewer is asking for the unbiased clustering of the scRNAseq dataset and is concerned that we may be using cell state rather than cell type for clustering the foxm1+ cells.*
We are now providing the full dataset of differentially expressed genes for each cluster against the rest of the dataset (Supplementary Table 2). Whilst we agree with the reviewer that the *foxm1*+ cluster is enriched with cell cycle – related genes, GO analysis also show over-representation of terms associated with signalling, Development and Neurogenesis (Fig 3J).

5- S/he wants us to exclude other possibilities for our observation that there is an increased number of progenitors and a decreased number of neurons in our mutant compared to wildtype.
We have shown now that neuronal progenitors are not over proliferating and we show evidence of a very low level (and no difference depending on genotype) of apoptosis in the regenerating spinal

cord (Appendix Figure S3). Altogether, these data support very strongly our hypothesis that it is the fate of dividing progenitors that changes in our mutant.

6- It will be helpful for the readers by showing the representative image of mty1 staining and to show the absolute number of counted cells in addition to percentages.
We are now showing images of Myt1 staining (Figure 5E). We have the absolute numbers if the reviewer wants them, but given the fact that we have an equivalent number of cells (DAPI) in wildtype and mutants (Figure EV5F), the difference only comes from the number of Myt1+ cells. The absolute number of Myt1+ cells show exactly the same result at the data normalised over DAPI.

9- *the reviewer suggests to delineate the transition from progenitor-to-neurons and the transition to foxm1+ cells*.
This is now shown on Figure EV4C-E. *S/he also asks for in vivo validation*. This will take a very long time from the identification of genes on the foxm1 path, followed by generating their knockout/knockdown and characterisation of the phenotype. Whilst we agree with the reviewer that this is an interesting direction of research, that we feel it is outside the scope of this manuscript.

10- *The authors should also take advantage of their foxm1-null frog line to compare the scRNA-seq data between wildtype and foxm1-null tadpoles during tail regeneration.*
We agree with the reviewer but this will be quite an undertaking (more than 60 amputations, genotyping in 2 days before spinal cord dissection) and a very expensive experiment. We think that at this point, it is outside the scope of this manuscript.

Minor comments

- *In order to make a strong statement on the "spinal cord-only" expression of Foxm1, Foxm1 ISH or IHC on the cross-section of the regenerate is appreciated (Figure1E).* This has been added on Figure 1E
- *The statistics is missing in Figure1F*: the statistics are now shown
- *Figure3F and Figure 4D require more explanation or the figures have to be simplified*: we have added explanation to Figure 3F (now Figure 4B) and explain better the method for Figure 4D (now Figure 5D).
- *It was not mentioned how CRISPR/Cas9 knock-down (Figure4A) was performed. Whether the term "knock-down" means mosaic F0 or heterozygous F1, is unclear.* We have now clarified this point: knock-down means crispant mosaic F0 or morpholino injection.
- *The quality of IF images should be improved (Figure4B*) This has been done throughout the manuscript (see my comments above)
- *Figure legends of the supplementary Figure1E-I are missed labeled to FigureS2I-E.* These mistakes have been corrected in the version of these figures.

1st Revision - Editorial Decision 23rd Jun 2021

Dear Dr. Dorey

Thank you for the submission of your revised manuscript. We have now received the enclosed reports from the referees, and I am happy to say that all support the publication of your study now. Referee 2 only has a minor suggestion that I would like you to incorporate before we can proceed with the official acceptance of your manuscript.

A few other editorial changes will also be required:

- A callout to Fig 3A and Fig EV3A is missing. The Appendix Figs S1+S3 panels are not called out. Please correct.

- The 2 Tables should be called Dataset EV1 and Dataset EV2, and the legends need to be added to the files. Please also correct the callouts in the manuscript text.

- Please add a table of content with page numbers to the Appendix file.

EMBO press papers are accompanied online by A) a short (1-2 sentences) summary of the findings and their significance, B) 2-3 bullet points highlighting key results and C) a synopsis image that is exactly 550 pixels wide and 200-600 pixels high (the height is variable). You can either show a model or key data in the synopsis image. Please note that text needs to be readable at the final size. Please send us this information along with the revised manuscript.

I would like to suggest a few changes to the abstract that needs to be written in present tense. Please let me know whether you agree with this:

Xenopus tadpoles have the ability to regenerate their tails upon amputation. Although some of the molecular and cellular mechanisms that globally regulate tail regeneration have been characterised, tissue-specific response to injury remains poorly understood. Using a combination of bulk and single cell RNA sequencing on isolated spinal cords before and after amputation, we identify a number of genes specifically expressed in the spinal cord during regeneration. We show that Foxm1, a transcription factor known to promote proliferation, is essential for spinal cord regeneration. Surprisingly, Foxm1 does not control the cell cycle length of neuronal progenitors but regulates their fate after division. In foxm1-/- tadpoles, we observe a reduction in the number of neurons in the regenerating spinal cord, suggesting that neuronal differentiation is necessary for the regenerative process. Altogether, our data uncover a spinal cord - specific response to injury and reveal a new role for neuronal differentiation during regeneration.

I look forward to seeing a final version of your manuscript as soon as possible. Please use this link to submit your revision: https://embor.msubmit.net/cgi-bin/main.plex

Kind regards,
Esther

Esther Schnapp, PhD
Senior Editor
EMBO reports

Referee #1:

The authors have carefully revised the manuscript, adding a significant amount of new data and comprehensively dealing with/addressing all points of criticism.

Referee #2:

The main objective of this manuscript is to investigate key factors that regulate the spinal cord regeneration in Xenopus tropicalis. The authors elegantly identified the spinal cord regeneration-specific upregulation of foxm1 by bulk RNA-sequencing and determined that ROS as an upstream of foxm1 pathway. scRNA-seq analysis revealed foxm1+ve population emerged in 3dpa tail regenerate and is associated with cell cycle-related genes. Interestingly, foxm1-KO does not affect the length of cell cycle. Phenotypic analysis on foxm1 knock-out/knock-down animals showed that foxm1 depletion in Xenopus tropicalis does not affect the proliferation of neuroprogenitors during embryogenesis and the foxm1-null tropicalis develop normally. However, after tail amputation foxm1 mutants showed impaired regeneration measured by length. The authors then attributed the phenotype to the change of progenitor orientation and the failure of progenitor-to-neuron transition.

I appreciate authors' effort to address most of my precious comments, including the characterization of the mutant lines, determining overall cell cycle length, scRNA-seq batch correction, improving image quality, and so on. Overall, I find the revised manuscript provides a convincing story.

A minor comment is that the authors use "dividing" and "proliferating" interchangeably in line 2, line 6 and line 9 of page 13. If I understand it correctly, the assumption was drawn from the conclusion that Tc was 50 hours (around 2 days), so a 2-day chase should encompass one cell cycle. However, this was not immediately apparent to me. The authors could improve the clarity in this paragraph.

Referee #3:

The new version of the Pelzer et al. manuscript includes the additional experiments that I suggested in the first review of the manuscript. These data reinforce the conclusions of the paper which is now appropriate for publication.

MANCHESTER 1824
The University of Manchester

Dr Karel Dorey
Lecturer
The University of Manchester
Faculty of Biology, Medicine and Health
Oxford Road
M13 9PT, Manchester, UK
Tel: 00-44-161 275 5319
Email: karel.dorey@manchester.ac.uk

Manchester, 24th of June 2021

Re: EMBOR-2020-50932V1

Dear Dr Schnapp,

We are very pleased that the reviewers thought that our manuscript entitled *Foxm1 regulates neural progenitor fate during spinal cord regeneration* by Pelzer *et al.* is suitable for publication in *EMBOreports.*

We have incorporated all the changes that you and referee two asked for. In the case of referee two's comment, whilst I understood what they were asking (I think), I could not see where we used dividing / proliferating in the page / lines mentioned. However, I have modified the sentence on page 13 lane 10/11 as follow:
"Because we estimated the cell cycle to be approximately 2 days (Fig 2H), we labelled cycling cells with EdU at 3 dpa and determined their fate after one cell cycle at 5 dpa using immunofluorescence."

Finally, for consistency, we now refer NPCs as neural progenitor cells throughout the text as before we used both neuronal and neural.

Thank you very much for handling our manuscript

Yours truly,

Dr Karel Dorey

2nd Revision - Editorial Decision 1st Jul 2021

Dr. Karel Dorey
University of Manchester
Faculty of Biology, Medicine and Health
Michael Smith Building
Oxford Road
Manchester M13 9PT
United Kingdom

Dear Dr. Dorey,

I am very pleased to accept your manuscript for publication in the next available issue of EMBO reports. Thank you for your contribution to our journal.

At the end of this email I include important information about how to proceed. Please ensure that you take the time to read the information and complete and return the necessary forms to allow us to publish your manuscript as quickly as possible.

As part of the EMBO publication's Transparent Editorial Process, EMBO reports publishes online a Review Process File to accompany accepted manuscripts. As you are aware, this File will be published in conjunction with your paper and will include the referee reports, your point-by-point response and all pertinent correspondence relating to the manuscript.

If you do NOT want this File to be published, please inform the editorial office within 2 days, if you have not done so already, otherwise the File will be published by default [contact: emboreports@embo.org]. If you do opt out, the Review Process File link will point to the following statement: "No Review Process File is available with this article, as the authors have chosen not to make the review process public in this case."

Should you be planning a Press Release on your article, please get in contact with emboreports@wiley.com as early as possible, in order to coordinate publication and release dates.

Thank you again for your contribution to EMBO reports and congratulations on a successful publication. Please consider us again in the future for your most exciting work.

Best regards,
Esther

Esther Schnapp, PhD
Senior Editor
EMBO reports

****

THINGS TO DO NOW:

You will receive proofs by e-mail approximately 2-3 weeks after all relevant files have been sent to our Production Office; you should return your corrections within 2 days of receiving the proofs.

Please inform us if there is likely to be any difficulty in reaching you at the above address at that time. Failure to meet our deadlines may result in a delay of publication, or publication without your corrections.

All further communications concerning your paper should quote reference number EMBOR-2020-50932V3 and be addressed to emboreports@wiley.com.

Should you be planning a Press Release on your article, please get in contact with emboreports@wiley.com as early as possible, in order to coordinate publication and release dates.

EMBO PRESS

YOU MUST COMPLETE ALL CELLS WITH A PINK BACKGROUND 🡻

PLEASE NOTE THAT THIS CHECKLIST WILL BE PUBLISHED ALONGSIDE YOUR PAPER

Corresponding Author Name: Karel Dorey

Journal Submitted to: EMBO reports
Manuscript Number: EMBOR-2020-50932V1

Reporting Checklist For Life Sciences Articles (Rev. June 2017)

This checklist is used to ensure good reporting standards and to improve the reproducibility of published results. These guidelines are consistent with the Principles and Guidelines for Reporting Preclinical Research issued by the NIH in 2014. Please follow the journal's authorship guidelines in preparing your manuscript.

A- Figures
1. Data
The data shown in figures should satisfy the following conditions:

- the data were obtained and processed according to the field's best practice and are presented to reflect the results of the experiments in an accurate and unbiased manner.
- figure panels include only data points, measurements or observations that can be compared to each other in a scientifically meaningful way.
- graphs include clearly labeled error bars for independent experiments and sample sizes. Unless justified, error bars should not be shown for technical replicates.
- if n< 5, the individual data points from each experiment should be plotted and any statistical test employed should be justified
- Source Data should be included to report the data underlying graphs. Please follow the guidelines set out in the author ship guidelines on Data Presentation.

2. Captions
Each figure caption should contain the following information, for each panel where they are relevant:

- a specification of the experimental system investigated (eg cell line, species name).
- the assay(s) and method(s) used to carry out the reported observations and measurements
- an explicit mention of the biological and chemical entity(ies) that are being measured.
- an explicit mention of the biological and chemical entity(ies) that are altered/varied/perturbed in a controlled manner.

- the exact sample size (n) for each experimental group/condition, given as a number, not a range;
- a description of the sample collection allowing the reader to understand whether the samples represent technical or biological replicates (including how many animals, litters, cultures, etc.).
- a statement of how many times the experiment shown was independently replicated in the laboratory.
- definitions of statistical methods and measures:
  - common tests, such as t-test (please specify whether paired vs. unpaired), simple χ2 tests, Wilcoxon and Mann-Whitney tests, can be unambiguously identified by name only, but more complex techniques should be described in the methods section;
  - are tests one-sided or two-sided?
  - are there adjustments for multiple comparisons?
  - exact statistical test results, e.g., P values = x but not P values < x;
  - definition of 'center values' as median or average;
  - definition of error bars as s.d. or s.e.m.

Any descriptions too long for the figure legend should be included in the methods section and/or with the source data.

In the pink boxes below, please ensure that the answers to the following questions are reported in the manuscript itself. Every question should be answered. If the question is not relevant to your research, please write NA (non applicable). We encourage you to include a specific subsection in the methods section for statistics, reagents, animal models and human subjects.

USEFUL LINKS FOR COMPLETING THIS FORM

http://www.antibodypedia.com
http://1degreebio.org
http://www.equator-network.org/reporting-guidelines/improving-bioscience-research-repor
http://grants.nih.gov/grants/olaw/olaw.htm
http://www.mrc.ac.uk/Ourresearch/Ethicsresearchguidance/Useofanimals/index.htm
http://ClinicalTrials.gov
http://www.consort-statement.org
http://www.consort-statement.org/checklists/view/32-consort/66-title
http://www.equator-network.org/reporting-guidelines/reporting-recommendations-for-tumo
http://datadryad.org
http://figshare.com
http://www.ncbi.nlm.nih.gov/gap
http://www.ebi.ac.uk/ega
http://biomodels.net/
http://biomodels.net/miriam/
http://jjj.biochem.sun.ac.za
http://oba.od.nih.gov/biosecurity/biosecurity_documents.html
http://www.selectagents.gov/

B- Statistics and general methods

Please fill out these boxes 🡻 (Do not worry if you cannot see all your text once you press return)

1.a. How was the sample size chosen to ensure adequate power to detect a pre-specified effect size?	sample size were not predetermined for a specific effect size
1.b. For animal studies, include a statement about sample size estimate even if no statistical methods were used.	For most experiments, at least three tadpoles from three different matings were analysed. For experiments where less than nine tadpoles were analysed, one to three tadpole(s) were used from three independent matings.
2. Describe inclusion/exclusion criteria if samples or animals were excluded from the analysis. Were the criteria pre-established?	no samples or animals were excluded from our analyses
3. Were any steps taken to minimize the effects of subjective bias when allocating animals/samples to treatment (e.g. randomization procedure)? If yes, please describe.	For regeneration experiments: the individuals were genotyped after the end of the experiment to avoid any bias in measuring re-growth. For quantification from immunofluorescence data, the images were randomised using a blind macro from FIJI and unblinded at the end of the quantification.
For animal studies, include a statement about randomization even if no randomization was used.	no randomization was used
4.a. Were any steps taken to minimize the effects of subjective bias during group allocation or/and when assessing results (e.g. blinding of the investigator)? If yes please describe.	genotypes were only revealed at the end of the experiments and images were randomised in FIJI
4.b. For animal studies, include a statement about blinding even if no blinding was done	For regeneration experiments: the individuals were genotyped after the end of the experiment to avoid any bias in measuring re-growth. For quantification from immunofluorescence data, the images were randomised using a blind macro from FIJI and unblinded at the end of the quantification.
5. For every figure, are statistical tests justified as appropriate?	yes
Do the data meet the assumptions of the tests (e.g., normal distribution)? Describe any methods used to assess it.	normal distribution was tested using a Shapiro-Wilk test in all experiments
Is there an estimate of variation within each group of data?	no

Is the variance similar between the groups that are being statistically compared?	N/A

C- Reagents

6. To show that antibodies were profiled for use in the system under study (assay and species), provide a citation, catalog number and/or clone number, supplementary information or reference to an antibody validation profile. e.g., Antibodypedia (see link list at top right), 1DegreeBio (see link list at top right).	anti-Myt1 (gift from Nancy Papalopulu), anti-Sox3 (gift from Nancy Papalopulu), anti-pH3 (06-570, Sigma) and anti-BrdU (clone MoBU-1, B35128, Invitrogen): Thuret et al. 2015 (doi:10.1242/bio.013391) - anti-acetylated tubulin (clone 6-11B1, Sigma) Panagiotaki et al. 2010 (doi: 10.1242/dev.053173) , anti-cleaved caspase 3 (9661, Cell Signalling Technology) was validated in this study and anti-PCNA ((PC10, Sigma) was used previously in Xenopus: Chernoff et al. 2018 (10.3389/fncel.2018.00045).
7. Identify the source of cell lines and report if they were recently authenticated (e.g., by STR profiling) and tested for mycoplasma contamination.	N/A

* for all hyperlinks, please see the table at the top right of the document

D- Animal Models

8. Report species, strain, gender, age of animals and genetic modification status where applicable. Please detail housing and husbandry conditions and the source of animals.	Xenopus tropicalis wild type and Crispr-induced mutant in the foxm1 locus. Frogs were either raised in the facility (some of the wild type and whole of the mutants) or obtained from the European Xenopus Research Centre (EXRC). The frogs are aged between 1 and 6 years old when used. The tadpoles analysed in this study were a mix of males and females. Up to 15 adult males and 10 females are housed in 23.3L tanks. They are rested for at least three months between use.
9. For experiments involving live vertebrates, include a statement of compliance with ethical regulations and identify the committee(s) approving the experiments.	All experiments were approved by a Home Office Licence (PFDA14F2D) and the local Animal Welfare and Ethical Review Body (AWERB).
10. We recommend consulting the ARRIVE guidelines (see link list at top right) (PLoS Biol. 8(6), e1000412, 2010) to ensure that other relevant aspects of animal studies are adequately reported. See author guidelines, under 'Reporting Guidelines'. See also: NIH (see link list at top right) and MRC (see link list at top right) recommendations. Please confirm compliance.	Reporting of animal experiments comply with guidelines

E- Human Subjects

11. Identify the committee(s) approving the study protocol.	N/A
12. Include a statement confirming that informed consent was obtained from all subjects and that the experiments conformed to the principles set out in the WMA Declaration of Helsinki and the Department of Health and Human Services Belmont Report.	N/A
13. For publication of patient photos, include a statement confirming that consent to publish was obtained.	N/A
14. Report any restrictions on the availability (and/or on the use) of human data or samples.	N/A
15. Report the clinical trial registration number (at ClinicalTrials.gov or equivalent), where applicable.	N/A
16. For phase II and III randomized controlled trials, please refer to the CONSORT flow diagram (see link list at top right) and submit the CONSORT checklist (see link list at top right) with your submission. See author guidelines, under 'Reporting Guidelines'. Please confirm you have submitted this list.	N/A
17. For tumor marker prognostic studies, we recommend that you follow the REMARK reporting guidelines (see link list at top right). See author guidelines, under 'Reporting Guidelines'. Please confirm you have followed these guidelines.	N/A

F- Data Accessibility

18: Provide a "Data Availability" section at the end of the Materials & Methods, listing the accession codes for data generated in this study and deposited in a public database (e.g. RNA-Seq data: Gene Expression Omnibus GSE39462, Proteomics data: PRIDE PXD000208 etc.) Please refer to our author guidelines for 'Data Deposition'.<br><br>Data deposition in a public repository is mandatory for:<br>a. Protein, DNA and RNA sequences<br>b. Macromolecular structures<br>c. Crystallographic data for small molecules<br>d. Functional genomics data<br>e. Proteomics and molecular interactions	raw data from bulk RNAseq and single cell RNAseq were deposited on ArrayExpress (https://www.ebi.ac.uk/arrayexpress/) with the following accession numbers: E-MTAB-8785 (for bulk experiments) and E-MTAB-8839 for single cell RNAseq
19. Deposition is strongly recommended for any datasets that are central and integral to the study; please consider the journal's data policy. If no structured public repository exists for a given data type, we encourage the provision of datasets in the manuscript as a Supplementary Document (see author guidelines under 'Expanded View' or in unstructured repositories such as Dryad (see link list at top right) or Figshare (see link list at top right).	
20. Access to human clinical and genomic datasets should be provided with as few restrictions as possible while respecting ethical obligations to the patients and relevant medical and legal issues. If practically possible and compatible with the individual consent agreement used in the study, such data should be deposited in one of the major public access-controlled repositories such as dbGAP (see link list at top right) or EGA (see link list at top right).	
21. Computational models that are central and integral to a study should be shared without restrictions and provided in a machine-readable form. The relevant accession numbers or links should be provided. When possible, standardized format (SBML, CellML) should be used instead of scripts (e.g. MATLAB). Authors are strongly encouraged to follow the MIRIAM guidelines (see link list at top right) and deposit their model in a public database such as Biomodels (see link list at top right) or JWS Online (see link list at top right). If computer source code is provided with the paper, it should be deposited in a public repository or included in supplementary information.	

G- Dual use research of concern

22. Could your study fall under dual use research restrictions? Please check biosecurity documents (see link list at top right) and list of select agents and toxins (APHIS/CDC) (see link list at top right). According to our biosecurity guidelines, provide a statement only if it could.	N/A
